# Statistical analysis of repertoire data demonstrates the influence of microhomology in V(D)J recombination

**DOI:** 10.1093/nar/gkaf250

**Published:** 2025-04-02

**Authors:** Magdalena L Russell, Assya Trofimov, Philip Bradley, Frederick A Matsen IV

**Affiliations:** Computational Biology Program, Fred Hutchinson Cancer Center, Seattle, WA 98109, United States; Molecular and Cellular Biology Program, University of Washington, Seattle, WA 98195, United States; Computational Biology Program, Fred Hutchinson Cancer Center, Seattle, WA 98109, United States; Department of Physics, University of Washington, Seattle, WA 98195, United States; Computational Biology Program, Fred Hutchinson Cancer Center, Seattle, WA 98109, United States; Institute for Protein Design, Department of Biochemistry, University of Washington, Seattle, WA 98195, United States; Computational Biology Program, Fred Hutchinson Cancer Center, Seattle, WA 98109, United States; Department of Genome Sciences, University of Washington, Seattle, WA 98195, United States; Department of Statistics, University of Washington, Seattle, WA 98195, United States; Howard Hughes Medical Institute, Seattle, WA 98195, United States

## Abstract

V(D)J recombination generates the diverse B and T cell receptors essential for recognizing a wide array of antigens. This diversity arises from the combinatorial assembly of V(D)J genes and the junctional deletion and insertion of nucleotides. While previous *in vitro* studies have shown that microhomology—short stretches of sequence homology between gene ends—can bias the recombination process, the extent of microhomology’s impact *in vivo*, particularly in humans, remains unknown. In this paper, we assess how germline-encoded microhomology influences trimming and ligation during V(D)J recombination using statistical inference on previously published high-throughput TCRα repertoire sequencing data. We find that microhomology increases both trimming and ligation probabilities, making it an important predictor of recombination outcomes. These effects are consistent across other receptor loci and sequence types. Further, we demonstrate that accounting for germline microhomology effects significantly alters sequence annotation probabilities and rankings, highlighting its practical importance for accurately inferring the V(D)J recombination events that generated an observed sequence. Together, these results enhance our understanding of how germline-encoded microhomologous nucleotides shape the human V(D)J recombination process.

## Graphical Abstract

**Graphical Abstract:**
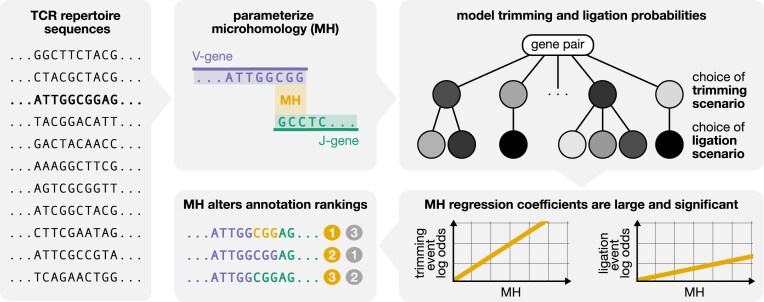

## Introduction

V(D)J recombination is an essential process for generating diverse B cell receptors and T cell receptors (TCRs). In this process, single V-, D- (if present), and J-genes are randomly selected from a pool of germline gene segments, then edited and joined together to form a uniquely recombined receptor sequence. Previous *in vitro* experiments have suggested that short stretches of sequence homology between gene ends, known as microhomology, can play a significant role in the V(D)J recombination process [1–9]. This raises the question of whether microhomology impacts V(D)J recombination *in vivo*, particularly in terms of recombination outcomes in humans with intact recombination machinery. Understanding this has practical implications for V(D)J recombination sequence *annotation*. Annotation means inferring the specific V(D)J recombination editing and joining processes that produced each sequence, forming the basis for many downstream B cell and T cell repertoire analyses. In this paper, we use statistical inference on high-throughput human TCR repertoire data to assess how microhomology influences various steps of the V(D)J recombination process.

In order to more fully set the stage, we will now summarize the relevant biological context. V(D)J recombination begins when the recombination activating gene (RAG) protein complex aligns two randomly chosen genes, removes the intervening chromosomal DNA between the two genes, and forms a hairpin loop at the end of each gene [10–12]. Each hairpin loop is then nicked open by the Artemis:DNA–PKcs complex [12–14]. Hairpin opening most frequently occurs at position +2, where position 0 refers to the edge of the hairpin and position -1 refers to the last nucleotide on the 5’ strand [13]; however, other hairpin opening positions are also possible [13, 14]. The Ku heterodimer (Ku70/Ku80) can bind to each nicked gene end and recruit non-homologous end joining factors, in any order, to repair the double stranded break [4, 5, 15]. From here, it is likely that the processing of the two gene ends occurs iteratively, with multiple rounds of action by a nuclease, polymerase, and ligase which eventually leads to a joining event to combine the two gene fragments [5, 9].

The various possible processing steps involved in this iterative end-joining stage are as follows. Nucleotides can be trimmed from each gene end through a mechanism suggested to involve the Artemis nuclease [6, 16–24]. Nucleotide deletion is thought to occur in a sequence-dependent fashion; for example, sequences with high AT content have been found to experience greater nucleotide loss than those with high GC content [3, 17–19], and the extent of deletions has been shown to depend on local nucleotide identity [25, 26], as well as sequence breathing capacity and length [26]. Additionally, non-template-encoded nucleotides, known as N-insertions, can be added by terminal deoxynucleotidyl transferase (TdT) [27–29]. TdT has a bias for the addition of purine-purine and pyrimidine-pyrimidine di-nucleotides suggesting that nucleotide addition depends on the previous addition [3, 25]. Further, nucleotide addition lengths and composition have been shown to depend on the presence (or absence) of nucleotide trimming at the gene ends [30]. Joining of the two gene ends is then carried out by XRCC4:DNA ligase IV, a flexible ligase that can ligate across gaps and incompatibilities between the ends, along with additional end-joining factors like XLF and PAXX that stabilize the ends, and polymerases that fill in gaps [7, 31–34].

The presence of microhomology, while not required, has been suggested to bias the outcome of the random V(D)J recombination processing steps. Microhomology can occur in several forms: (1) **terminal microhomology**, found at the ends of genes prior to trimming/insertion and encoded in the germline. [While we use this term to describe germline-encoded microhomologous nucleotides located at gene ends (prior to trimming), other sources [5, 6] often use the term more broadly to describe all microhomologous nucleotides located at gene ends, including both germline-encoded nucleotides and those generated through N-insertion.]; (2) **interior microhomology**, located *within* the sequences and also germline-encoded; and (3) **insertion-dependent microhomology**, created by N-insertions and not encoded in the germline. Because terminal and interior microhomology are both germline-encoded, we will collectively refer to them as *germline-encoded microhomology*. If present, terminal microhomology can directly guide ligation without additional processing. In contrast, interior and insertion-dependent microhomology may necessitate deletions or further N-insertions before microhomology-mediated ligation can occur. This paper will focus exclusively on germline-encoded microhomologies, excluding insertion-dependent microhomology.

**Figure 1.**
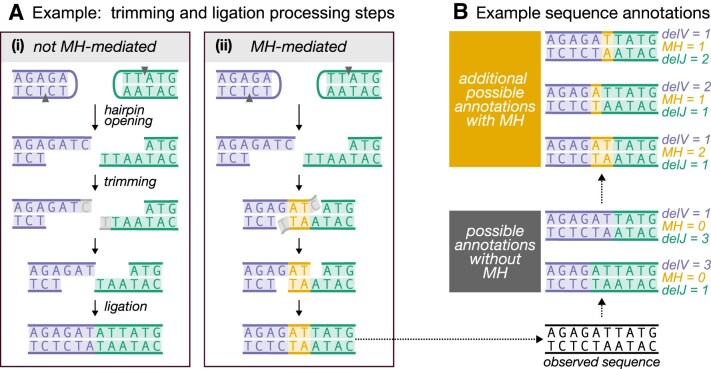
(**A**) Illustration of how germline-encoded microhomology (MH) could affect trimming and/or ligation during V(D)J recombination. We use sequences without N-insertions to quantify these effects, leveraging germline V- and J-gene sequences to identify potential MH-mediated ligation events. The example shows germline-encoded interior microhomologous regions (yellow) and trimmed nucleotides (grey) for a V-gene (purple, left sequence) and J-gene (green, right sequence), highlighting two regimes: (i) no MH influence and (ii) MH-mediated trimming and ligation. In these scenarios, germline-encoded microhomologous regions are classified as terminal microhomology when they facilitate the ligation of untrimmed sequences, and as interior microhomology when they facilitate the ligation of trimmed sequences. MH could affect trimming, ligation, or both, leading to distinct sequences regardless of whether the genes are trimmed equally or differently. We illustrate how other forms of microhomology could affect trimming and ligation during V(D)J recombination within Supplementary Fig. S1. (**B**) Illustration of possible V(D)J recombination annotations for sequences lacking N-insertions that potentially ligate with MH. Existing annotation software does not account for MH and assigns shared nucleotides to only one sequence (grey box annotations). However, additional annotations that incorporate MH (yellow box) are possible but are not considered by existing software. Example trimming scenarios, given by delV and delJ, and ligation scenarios, given by MH, for each possible annotation are shown. Our modeling aims to consider all annotations, both with and without MH, for each sequence.

Experiments *in vitro* and with model organisms have suggested that microhomology (i.e. 1–4 nucleotides) is an important factor in V(D)J recombination. Although microhomology between gene ends is not essential for joining (Fig. 1A part (i)) [35, 36], it has been shown to improve joining efficiency and bias the outcome towards using the microhomologous region to guide trimming and ligation (Fig. 1A part (ii)) [3–9]. For example, reconstitution experiments suggest that sequences with microhomology can stabilize gene ends without requiring additional end-joining factors like XLF and PAXX and germline-encoded microhomology may reduce the necessity for template-independent addition by polymerase-μ and TdT [7], possibly explaining the enhanced ligation efficiency. *In vitro* studies show that 1 or 2 nucleotides of germline-encoded microhomology are present in nearly 60% of ligated coding joints in the absence of TdT [3], with similar observations reported in neonatal mice when TdT levels are low [1, 2]. However, this frequency drops substantially when TdT is present, as TdT-mediated additions are thought to create stronger insertion-dependent microhomology [3, 5, 6]. The involvement of microhomology in ligation appears to be more complex when it is not present at sequence ends or generated through nucleotide addition. Most gene ends lack terminal microhomology after hairpin opening but share interior microhomology [7, 8, 14]. In such cases, the Artemis–DNA-PKcs complex has been shown to trim gene ends to expose interior microhomology (Fig. 1 A part(ii)) [7, 8]. Supplementary Fig. S1 provides an extended overview illustrating how different forms of microhomology could influence V(D)J recombination.

This essential biochemical work has demonstrated that microhomology can significantly affect V(D)J recombination; however, it does not demonstrate its importance for shaping V(D)J recombination in humans. In addition to being an issue of intrinsic interest, the role of microhomology has practical implications as well: if microhomology impacts the probability of V(D)J recombination annotations (i.e. numerical histories of recombination events such as gene choice, trimming, insertion, ligation, etc.), then corresponding terms should be incorporated into software that infers recombination probabilities. This would ensure that additional annotations involving microhomology are also considered (Fig. 1B).

Statistical inference on high-throughput repertoire sequencing datasets allows exploration of the *in vivo* V(D)J recombination mechanism in humans. In fact, existing probabilistic models of V(D)J recombination, such as IGoR [37], have provided interesting and important insights about the natural underlying mechanism by learning statistics of V(D)J recombination. These models have revealed significant dependencies between recombination events, such as gene usage and trimming, and have provided estimates of the overall probabilities of generating specific TCR sequences, thereby helping to disambiguate the effects of generation from selection [25, 37]. Similar statistical approaches have been successfully applied to understand the sequence-dependent process of nucleotide trimming, revealing significant connections between trimming patterns and local sequence identity, length, and wider GC content [26]. However, to our knowledge, no probabilistic models of V(D)J recombination incorporating microhomology have been developed.

In this paper, we explore the extent to which germline-encoded microhomology biases trimming and ligation during V(D)J recombination using statistical inference on high-throughput TCRα repertoire sequencing data [38, 39]. We have designed a flexible probabilistic modeling framework, allowing us to quantify the extent to which germline-encoded microhomology biases trimming and ligation probabilities. Our results show that the presence of germline-encoded microhomology significantly increases trimming and ligation probabilities, and is an important predictor of the choices made in these processes. These observations are consistent with sequences from an independent TCRα validation dataset, as well as with sequences from other receptor loci such as TCRγ. Additionally, we demonstrate that explicitly including microhomology-related terms in our model substantially impacts sequence annotation probabilities and overall V(D)J recombination annotation rankings. Together, these findings enhance our understanding of the involvement of germline-encoded microhomology in the V(D)J recombination process and highlight the importance of accounting for microhomology-related effects in receptor sequence processing and analysis.

## Materials and methods

### Terminology

In this paper, we investigate the mechanisms of trimming and ligation as they occur between V- and J-gene pairs during V(D)J recombination. We will use these terms throughout the paper:

- **Trimming scenario**: A specific pair of trimming events, one at the V-gene end and one at the J-gene end.
- **Ligation scenario**: A specific number of germline-encoded microhomologous nucleotides shared between the trimmed V-gene and J-gene, facilitating their ligation. The possible ligation scenarios for a given V-J gene pair are determined by their germline sequences and the extent of trimming.
- **Joint trimming and ligation scenario probability**: The normalized probability of a particular combination of trimming and ligation scenarios occurring for a V-J gene pair, considering all possible trimming-ligation combinations for that pair.
- **V(D)Jrecombination annotation**: A specific set of V(D)J recombination events that produce a sequence, including trimming, insertion, and ligation scenarios.
- **V(D)Jrecombination annotation probability**: The normalized probability of a particular V(D)J recombination annotation for an observed sequence, calculated from all possible annotations for that sequence. We restrict our analysis to sequences without N-insertions such that we can derive these probabilities from joint trimming and ligation scenario probabilities and normalize over all possible scenario combinations for that specific observed sequence.

### Data and data processing overview

To explore trimming and ligation patterns, we analyzed TCRα-immunosequencing data from 10 individuals [38, 39]. The *TRA* locus was chosen for its higher sequence diversity between joining genes (V- and J-gene pairs) compared to the *TRB* locus.

We used the IGoR software (version 1.4.0), designed to learn unbiased recombination statistics from immune sequence reads [37], to infer possible V(D)J recombination annotations and their associated likelihoods for each sequence. Each annotation consists of inferred V- and J-gene assignments, trimming lengths, and the number of N-insertions. For each sequence, we processed these annotations in two steps. First, we sampled a single V- and J-gene assignment and N-insertion amount based on their posterior probabilities. Sequences with N-insertions were excluded to focus on germline-microhomology-mediated ligation events, as N-insertions complicate ligation pattern analysis due to their unknown nucleotide composition prior to ligation and indicate that *germline*-microhomology-mediated ligation did not occur. In these training data, we found that roughly 5% of sequences contained zero inferred N-insertions.

Next, given the IGoR-inferred V- and J-gene assignments and N-insertion amounts, we determined the set of possible trimming and ligation scenarios for each sequence. Since IGoR does not account for microhomology and assigns shared nucleotides to only one sequence, we did not use the corresponding IGoR-inferred trimming annotation. Instead, we adapted this IGoR-inferred trimming annotation to account for germline-encoded microhomology. This approach allowed us to generate a set of possible trimming and ligation scenario annotations for each sequence, including those that involve germline-encoded microhomologous nucleotides (see Fig. 1B, Supplementary Fig. S12, and Supplementary Materials for details).

Additionally, TCR sequences can be categorized as “productive” if they code for a functional protein, or “non-productive” otherwise, arising from out-of-frame recombination or presence of stop codons. Each T cell can undergo recombination at two alleles; if the first is non-productive and the second successful, both sequences can be sequenced as part of the repertoire. Non-productive sequences do not generate proteins for thymic selection, and their recombination statistics should reflect only the V(D)J recombination process [25, 40, 41]. In contrast, productive sequence statistics reflect both recombination and selection. To study nucleotide trimming and ligation during V(D)J recombination without selection effects, we included only non-productive sequences in our training dataset. In these data, we found that roughly 67% of sequences were non-productive.

To validate our findings, we also analyzed productive sequences from the training dataset and both productive and non-productive sequences from independent TCRα-immunosequencing data from 10 healthy individuals [39] and TCRγ-immunosequencing data from 23 healthy bone marrow donors [42]. These validation datasets underwent the same IGoR-based annotation and filtering procedures as used for the training dataset.

Further details on these datasets and processing steps are provided in the Supplementary Materials.

### Modeling assumptions

We explore the impact of germline-encoded microhomology on V(D)J recombination by modeling the joint probability of trimming and ligation scenarios given V-gene and J-gene sequences. Our approach relies on the following biological assumptions:

- Nucleotide trimming precedes ligation [43]
- Each gene’s DNA hairpin is opened by a single-stranded break during the early stages of V(D)J recombination [3, 13, 14, 18, 19].
- This hairpin nick typically occurs at the +2 position, producing a 4-nucleotide 3’-overhang with two 3’-most nucleotides being considered P-nucleotides [13, 14]
- If any part of the original gene sequence is deleted, all P-nucleotides will also be deleted [3, 44].

To simplify our analysis, we consider only the “top” strand for V-genes (5’-to-3’) and the “bottom” strand for J-genes (3’-to-5’), consistent with the most common overhang polarities. Trimming is indexed from the 3’ end of each strand, with trimming sites corresponding to specific coding sequence positions. Supplementary Fig. S2 illustrates this sequence orientation along with the corresponding definitions.

### Notation and modeling set-up

In order to set up our model, we will now summarize relevant notation. We uniformly sample a sequence, *X*, from a TCRα repertoire of filtered sequences. The following variables are random due to the choice of *X*, but are deterministic given *X*, as they are determined by sampling from the recombination annotations inferred by IGoR based on their posterior probabilities. Let V and J be random variables representing the V-gene and J-gene, respectively, and I be a random variable representing the number of N-insertions. Let Q represent the productivity of the observed sequence, which can be either productive or non-productive. We define VJ as an ordered pair of IGoR-inferred genes: VJ = (V, J). Let MH be a random variable denoting the count of shared germline-encoded microhomologous nucleotides in the ligated sequence, and delV and delJ be random variables representing the number of nucleotides deleted from the V- and J-gene, respectively. Together, we define delVJ = (delV, delJ) as the pair of trimming lengths (a “trimming scenario”) and *M* as a “ligation scenario.”

For notational convenience we assume delV and delJ each take on an integer value on the interval [−2, …, 14], where values outside this range are considered nonsensical and assigned a probability of zero. Negative values indicate P-nucleotide deletions: a deletion of 0 means the deletion stops at the end of the germline gene sequence (e.g. two P-nucleotides are trimmed off), while a deletion of -2 indicates no deletion of P-nucleotides or gene sequence nucleotides. This indexing is consistent with the IGoR software [37] and illustrated in Supplementary Fig. S2B.

Existing annotation tools like IGoR [37] do not account for microhomology and attribute shared nucleotides to only one sequence when inferring trimming scenario annotations. Instead of using IGoR-inferred trimming annotations directly, we construct a set of possible trimming and ligation scenarios for each sequence, including those involving germline-encoded microhomologous nucleotides, based on the observed sequence and known germline gene sequences. As such, given a sequence *X* with gene pair VJ and zero N-insertions (I = 0), the set of possible trimming and ligation scenario annotations is described by combinations of delVJ and MH (as illustrated in Fig. 1 B). While both delVJ and MH can be considered random variables, they are dependent on one another—meaning the possible values of MH are constrained by delVJ and vice versa. The resulting set, *A*_*X*_, includes all feasible trimming and ligation scenarios consistent with *X*. This set is deterministic given *X*, but random due to the sampling of *X*. Details of the procedure to construct *A*_*X*_ are provided in the Supplementary Materials.

Our goal is to model trimming and ligation scenario probabilities given V- and J-gene pairs. To estimate the empirical conditional probability density function, let *C*(VJ, Q, I = 0) represent the count of TCRs within a sampled repertoire with productivity Q, using gene pair VJ, and with zero N-insertions. Let *C*(delVJ, MH, VJ, Q, I = 0) represent the count with trimming scenario delVJ, ligation scenario MH, zero N-insertions, productivity Q, and gene pair VJ. The empirical conditional probability density function is defined as:


\begin{eqnarray*}
P_\text{emp}(\mathrm{delVJ}, \mathrm{MH}\mid \mathrm{VJ}, \mathrm{Q}, \mathrm{I}=0) = \frac{C(\mathrm{delVJ}, \mathrm{MH}, \mathrm{VJ}, \mathrm{Q}, \mathrm{I}=0)}{C(\mathrm{VJ}, \mathrm{Q}, \mathrm{I}=0)}.
\end{eqnarray*}

To achieve our goal, we will train a conditional logit model, a type of logistic model designed to model discrete choices among multiple alternatives. Specifically, we aim to model *P*(delVJ, MH∣VJ, Q, I = 0) using sequence-level parameters, including those that capture germline-microhomology-related effects, with our TCRα repertoire training dataset. However, because the true trimming and ligation annotations (delVJ, MH) for each sequence are latent, we cannot directly compute this probability density. To address this challenge, we assign probabilities to each potential annotation based on model likelihoods. Since these probabilities depend on model parameters, we use an expectation-maximization algorithm for parameter inference, which we describe in detail in subsequent sections. We summarize all the notation discussed in this section, as well as in the following sections, in Supplementary Table S1.

### Model formulation

In our previous work, we established that local nucleotide identities at trimming sites (the “trimming motif”) and the counts of GC or AT nucleotides beyond these motifs (the “3’ base count” and “5’ base count”) are strong predictors of trimming probabilities for single gene sequences [26]. Building on this foundation, we have integrated these established model features with newly developed germline-microhomology-related features to assess their combined effects on trimming and ligation processes (see Supplementary Fig. S2 and Supplementary Materials for detailed definitions).

To this end, we developed a two-step conditional logit model to evaluate the joint probabilities of trimming and ligation scenarios for V- and J-gene pairs. The model describes a generative process in two steps:

- **Trimming scenario choice**: The probability *P*(delVJ∣VJ, Q, I = 0), of choosing a trimming scenario delVJ for a given V-J gene pair VJ, sequence productivity Q, and N-insertion amount I = 0. This choice is determined by the established “trimming motif,” “3’ base count,” and “5’ base count” parameters for each gene, in addition to a new parameter that quantifies the effect of germline-encoded microhomology on trimming. Specifically, this parameter measures the importance of the average number of germline-encoded microhomologous nucleotides between two trimmed sequences, a value that varies depending on the chosen trimming scenario. We denote the set of trimming-related parameters by $\boldsymbol{\beta }_\texttt {trim}$.
- **Ligation scenario choice**: The probability, *P*(MH∣delVJ, VJ, Q, I = 0), of choosing a ligation scenario MH for a given trimming scenario delVJ, V-J gene pair VJ, sequence productivity Q, and N-insertion amount I = 0. This choice is determined by a novel microhomology parameter related to ligation, which quantifies the importance of the number of germline-encoded microhomologous nucleotides that ultimately appear in the final ligated sequence. We denote this set of ligation-related parameters by $\boldsymbol{\beta }_\texttt {lig}$.

All of these modeling parameters are summarized in Supplementary Table S2, illustrated in Supplementary Fig. S2D, and described in detail in the Supplementary Materials.

The mental model of this two-step process is that trimming occurs first, independently of ligation, and then ligation occurs, conditioned on the trimming scenario. However, *P*(delVJ∣VJ, Q, I = 0) will be parameterized by both trimming- and ligation-related parameters ($\boldsymbol{\beta }_\texttt {trim}$ and $\boldsymbol{\beta }_\texttt {lig}$) because the model is conditioned on sequence productivity (Q), which is jointly determined by trimming and ligation. This dependency ensures that trimming probabilities properly account for how productivity constraints prune the space of possible ligation scenarios associated with each trimming scenario, correcting for any biases introduced by this non-uniform pruning (see Supplementary Materials for more details). Despite this dependency, the trimming-related parameters ($\boldsymbol{\beta }_\texttt {trim}$) and ligation-related parameters ($\boldsymbol{\beta }_\texttt {lig}$) are still designed to capture the distinct effects of various sequence-level features on trimming and ligation, respectively.

The joint probability of selecting a trimming scenario delVJ and a ligation scenario MH for a V-J gene pair VJ, sequence productivity Q, and zero N-insertions can thus be factored and modeled using trimming and ligation parameters $\boldsymbol{\beta }_\texttt {trim}$ and $\boldsymbol{\beta }_\texttt {lig}$ as:


\begin{eqnarray*}
&& P(\mathrm{delVJ}, \mathrm{MH}\mid \mathrm{VJ}, \mathrm{Q}, = 0; \boldsymbol{\beta }_\texttt {trim}, \boldsymbol{\beta }_\texttt {lig})\\ &&\quad :=\, P(\mathrm{delVJ}\mid \mathrm{VJ}, \mathrm{Q}, \mathrm{I}=0; \boldsymbol{\beta }_\texttt {trim}, \boldsymbol{\beta }_\texttt {lig})\\ &&\qquad \times P(\mathrm{MH}\mid \mathrm{delVJ}, \mathrm{VJ}, \mathrm{Q}, \mathrm{I}=0; \boldsymbol{\beta }_\texttt {lig}).
\end{eqnarray*}

**Figure 2.**
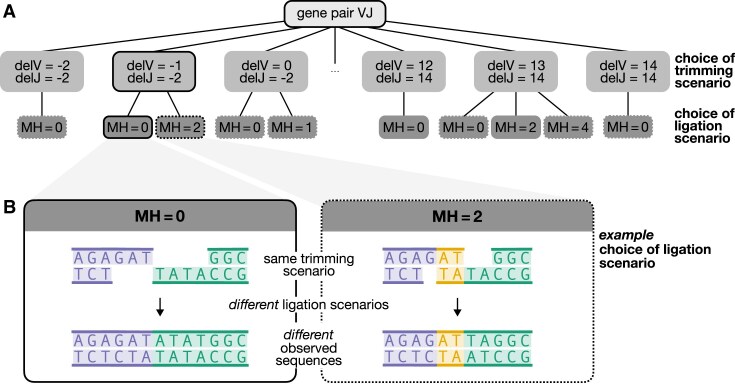
(**A**) Schematic of trimming and ligation choices for an arbitrary V-J gene pair, denoted by the random variable VJ. The first choice is the trimming scenario, represented by the random variable delVJ, which consists of a pair of V- and J-gene trimming amounts delV and delJ (e.g. each can range from −2 to 14 nucleotides). The next choice is the ligation scenario, represented by the random variable MH, which captures the number of germline-encoded microhomologous nucleotides used. The available ligation scenario choices depend on the germline sequences of the two genes being joined. Trimming and ligation scenarios resulting in productive and non-productive sequences are shown in solid and dashed boxes, respectively. (**B**) Illustration of the possible ligation scenarios for an example pair of trimmed sequences. The chosen ligation scenario affects the resulting observed sequence. The trimmed V-gene sequence is shown in purple (left sequence), the trimmed J-gene sequence in green (right sequence), and germline-encoded interior microhomologous nucleotides in yellow. Deletions are indexed such that a deletion of 0 corresponds to the end of the germline gene sequence (two P-nucleotides trimmed) and -2 corresponds to the full sequence (no P-nucleotides trimmed), as illustrated in Supplementary Fig. S2B. The ligation choices represented by MH = 0 and MH = 2 correspond to scenarios where zero or two germline-encoded microhomologous nucleotides (shown in yellow) are used to ligate the sequences, as reflected in the final observed sequence. Germline-encoded microhomologous regions are classified as terminal microhomology when they facilitate the ligation of untrimmed sequences, and as interior microhomology when they facilitate the ligation of trimmed sequences.

Fig. 2 illustrates the two-step structure of this model and the decision-making process for an example V-J gene pair.

These parameters, $\boldsymbol{\beta }_\texttt {trim}$ and $\boldsymbol{\beta }_\texttt {lig}$, are designed to quantify how sequence-level features, particularly germline-encoded microhomology, influence trimming and ligation choices during V(D)J recombination. Importantly, the magnitude of microhomology’s influence in guiding these choices is quantified by these conditional logit model parameters, highlighting its role in the recombination process. We validated the model’s ability to detect these effects through a series of simulations (see Supplementary Materials). In order to assess the significance of germline-microhomology-related terms in downstream analyses, such as in V(D)J recombination sequence annotation, we designed the model with the flexibility to include or exclude germline-microhomology-related parameters for both trimming and ligation decisions.

### Model training

We trained our conditional logit model using non-productive sequences without N-insertions and their corresponding sets of possible trimming and ligation scenarios (as described earlier). Training this model is complex because the true trimming and ligation scenarios for each sampled sequence are latent variables that depend on the model parameters. To estimate probabilities for each potential scenario, we assigned likelihoods based on our model and used an expectation-maximization (EM) algorithm for parameter inference.

Standard regression methods in R or Python could not support this type of optimization, so we implemented the EM algorithm using the JAX and JAXopt packages in Python, which support automatic differentiation [45, 46]. This algorithm converged within 25 iterations (Supplementary Fig. S3). Further details about the EM algorithm and model formulation are provided in the Supplementary Materials.

### Assessing significance of model parameters

When training our model, we infer a set of model parameters $\boldsymbol{\hat{\beta }} = \lbrace \boldsymbol{\hat{\beta }}_\texttt {trim}, \boldsymbol{\hat{\beta }}_\texttt {lig}\rbrace$ where $\boldsymbol{\beta }_\texttt {trim}$ are trimming-related parameters and $\boldsymbol{\beta }_\texttt {lig}$ are ligation-related parameters. Since the model is a conditional logit model, each parameter represents the change in the log_10_ odds of trimming and/or ligating at a specific scenario for a unit increase in the corresponding feature value, while holding all other features constant. To assess the significance of each individual parameter $\hat{\beta } \in \boldsymbol{\hat{\beta }}$, we test the null hypothesis that $\hat{\beta } = 0$. This approach enables us to understand the contribution of each parameter separately, allowing us to evaluate the impact of specific sequence features, such as the extent of germline-encoded microhomology, on the probability of recombination events.

To test significance, we estimate the standard error of each inferred parameter using a bootstrap method, with observed sequences as the sampling unit. For each bootstrap iteration, we sample sequences from the training dataset with replacement and train a new model to re-estimate the parameters. This process is repeated 1000 times, resulting in 1000 parameter estimates, which we use to calculate the standard error for each parameter. Using these standard errors, we calculate the test statistic:


\begin{eqnarray*}
T(\hat{\beta }) = \frac{\hat{\beta }}{\operatorname{se}(\hat{\beta })}.
\end{eqnarray*}

We compare $T(\hat{\beta })$ to a *N*(0, 1) distribution to obtain each p-value. We assess the significance of each model parameter using a Bonferroni-corrected threshold, adjusting for the total number of parameters being evaluated in the model.

### Validating model using likelihood ratio testing

To determine whether adding the germline-microhomology-related terms significantly improves our model’s fit to the observed data, we use a likelihood ratio test (LRT) to compare our full model that includes these terms to a simpler model that excludes them. This approach enables us to assess the collective impact of adding a set of parameters—in this case, the germline-microhomology-related parameters—to the model.

The LRT statistic compares the log-likelihoods of the two nested models:


\begin{eqnarray*}
\text{LR} = 2 \times (\mathcal {L}_{\texttt {MH}} - \mathcal {L}_{\texttt {noMH}}).
\end{eqnarray*}

Here, $\mathcal {L}_{\texttt {MH}}$ is the log-likelihood for the model with microhomology terms (defined in the Supplementary Materials, 20), while $\mathcal {L}_{\texttt {noMH}}$ is the log-likelihood for the simpler model without these terms. The LRT statistic approximately follows a chi-square distribution with degrees of freedom equal to the number of additional parameters in the more complex model (e.g. two germline-microhomology-related parameters in this case).

This test allows us to calculate a *P*-value for the likelihood ratio, which indicates whether the inclusion of germline-microhomology-related parameters significantly improves model fit. The LRT is particularly useful for evaluating the collective contribution of related parameters, rather than individual effects. While we use bootstrap testing to assess the significance of individual parameters (as described in the previous section), the LRT enables us to evaluate the combined impact of adding germline-microhomology-related terms, allowing us to determine whether these terms are collectively biologically meaningful in the context of the observed data.

## Results

### Germline-encoded microhomology significantly increases probabilities of both trimming and ligation events

Complementary sequence regions capable of forming microhomologous regions during V(D)J recombination are common between germline V- and J-genes in the *TRA* locus. The median average number of germline-encoded microhomologous nucleotides across the ensemble of possible trimming scenarios for these germline V- and J-gene pairs is 0.1978 (Supplementary Fig. S4). This median corresponds to 1.3149 possible ligation scenarios per trimming scenario (Supplementary Figs S5 and S6). Given that a median of exactly one ligation scenario per trimming scenario would indicate all V(D)J recombination annotations involve zero germline-encoded microhomology, this suggests that many trimming scenarios allow for multiple ligation outcomes, both with and without germline-encoded microhomology. Additionally, complementary sequence regions and their corresponding ligation scenario options are distributed across trimming scenarios depending on the specific V- and J-gene pair (Supplementary Figs S4 and S6). This distribution highlights the potential for both interior and terminal microhomology to influence trimming and ligation outcomes.

To quantify the effects of germline-encoded microhomologous nucleotides on trimming and ligation, we employed our model, which incorporates various sequence-level parameters, including those related to germline-encoded microhomology. We validated the model’s capability to detect germline-encoded microhomology effects through a series of simulations, designed to generate sequences by sampling trimming and ligation scenarios under different microhomology regimes: no germline-encoded microhomology effect, germline-encoded microhomology affecting either trimming or ligation choices exclusively, and germline-encoded microhomology influencing both. After training our model using each of these simulated datasets, we confirmed its sensitivity to detecting variable germline-encoded microhomology effects across different conditions (Supplementary Fig. S7).

**Figure 3.**
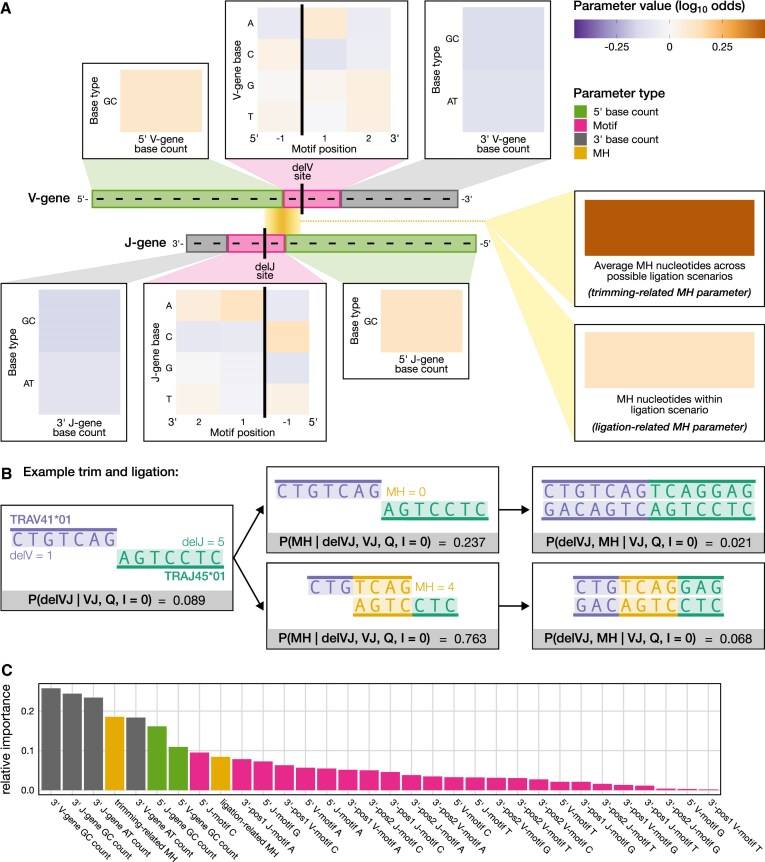
Although sequence-based parameters such as gene-specific trimming motifs and base counts contribute meaningfully to predicting trimming and ligation probabilities, the extent of germline-encoded microhomology (MH) between sequences exerts a strong effect, especially in increasing trimming probabilities. (**A**) Illustration of sequence features and their alignment with an arbitrary V- and J-gene pair at example trimming sites, which correspond to the number of nucleotides deleted from each gene (represented by the random variables delV and delJ), along with inferred model parameters. V- and J-gene trimming motif parameters (pink) reflect the influence of adjacent nucleotides on trimming probabilities. Trimming motif positions are indexed relative to the inferred trimming site for each gene, with negative indices indicating positions 5’ of the trimming site and positive indices indicating positions 3’. V- and J-gene base count parameters (green and grey) reflect the influence of upstream and downstream AT and GC nucleotide composition on trimming probabilities. Specifically, we find that an increase in GC nucleotides 5’ of the motif increases trimming probabilities, while an increase in AT or GC nucleotides 3’ of the motif decreases them. The model excludes 5’ AT nucleotide counts. MH between sequences (gold box) strongly influences both trimming and ligation probabilities, with a larger positive effect on trimming. Black vertical lines indicate example trimming sites. Each parameter represents the change in log_10_ odds of trimming or ligating due to an increase in the feature value, assuming all other features are held constant. (**B**) Our model demonstrates that increasing MH generally raises trimming and ligation probabilities, as shown in example scenarios for the most frequently used gene pair, TRAV41*01 (purple, left sequence) and TRAJ45*01 (green, right sequence). In the bottom row, four nucleotides of MH (gold) result in a most probable trimming and ligation scenario (left and middle boxes) with a joint probability of 0.068 (right box). In contrast, the top row shows the same trimmed sequences ligating with zero MH, leading to a lower joint probability of 0.021. Trimming and ligation probabilities are inferred across trimming scenarios (delVJ) and ligation scenarios (MH) for a V-J gene pair (VJ), yielding sequence productivity (Q) with zero N-insertions (I = 0). (**C**) Parameters for 3’ AT and GC base counts have the highest relative importance (grey), followed by the trimming-related microhomology parameter (yellow). Relative importance was calculated using a model trained with standardized features, where the absolute values of parameter estimates indicate their contribution to the model.

We then fit our model to the real TCRα training dataset to quantify the actual effects of germline-encoded microhomology, along with other sequence-level features, on the probabilities of trimming and ligation events. Since the model is a conditional logit model, each model parameter reflects the change in the log_10_ odds of trimming and/or ligating at a specific scenario due to an increase in the corresponding feature value, assuming all other features remain constant. We assessed the significance of each model parameter’s influence on trimming and ligation event probabilities by estimating their standard errors with bootstrap methods and applying a z-test to obtain a *P*-value (see “Materials and methods”). We used a Bonferroni-corrected significance threshold of 0.0016, adjusted for the total number of model parameters, and report parameters on the log_10_ scale. Our results indicate that the number of germline-encoded microhomologous nucleotides between two sequences substantially influences both trimming (parameter = 0.4484) and ligation (parameter = 0.1272) outcomes, with both effects being highly significant (*P*-values smaller than machine tolerance, *p* ≃ 0) (Fig. 3A).

This relationship is further demonstrated by notable increases in joint trimming and ligation probabilities for scenarios with more germline-encoded microhomology, as illustrated in Fig. 3B, which highlights two trimming and ligation scenarios from the most common V-J gene pair, TRAV41*01 and TRAJ45*01. While the influence of germline-encoded microhomology on trimming was stronger than on ligation, these effects appear to be interdependent. Interestingly, when training the model using sequences containing N-insertions (indicating a lack of ligation solely dependent on germline-encoded microhomology), germline-encoded microhomology had a small but significant effect on trimming probabilities (parameter = 0.0059; *P*-value smaller than machine tolerance, *p* ≃ 0) (Supplementary Fig. S8). The parameterization of sequence features in this case is described in the Supplementary Materials and illustrated in Supplementary Fig. S13. This model demonstrates that germline-encoded microhomology may independently influence trimming, suggesting a nuanced role beyond its interaction with ligation. However, it is possible that sequences containing N-insertions were ligated using microhomologous nucleotides derived from both N-insertions and germline-encoded regions, which could contribute to the observed trimming-related effects and complicate the interpretation of these signals.

Returning to the original model, in addition to germline-encoded microhomology effects, we identified significant “trimming motif”, “3’ base count”, and “5’ base count” parameters for the probabilities of both V- and J-gene trimming events. These parameters, previously introduced in our analyses of trimming patterns for single V- and J-gene sequences [26], showed results consistent with our previous work. As in our prior work, the local sequence context (“trimming motif”) for each gene was modeled using a position weight matrix from a three-nucleotide window around each trimming site. We observed similar patterns for both V-gene and J-gene local trimming contexts, where C and A nucleotides had the largest influence on trimming outcomes (Fig. 3A). The “5’ base count” and “3’ base count” parameters reflect how upstream and downstream AT and GC nucleotide composition influence trimming probabilities. These features are based on the raw counts of AT and GC nucleotides 5’ and 3’ of the trimming motif. The 5’ base count parameters act as a proxy for sequence-breathing effects, indicating a preference for GC content upstream of the motif. In contrast, the 3’ base count parameters capture two effects: the absolute position of the trimming site, as the total AT and GC counts downstream correspond to this position, and sequence-breathing effects driven by AT and GC content downstream of the motif. Our analysis showed that increasing GC nucleotides 5’ of the motif (which decreases sequence-breathing capacity) raised trimming probabilities. In contrast, increasing both AT and GC nucleotides 3’ of the motif (which increases absolute position) reduced trimming probabilities for both gene types (Fig. 3A).

Finally, we examined the relative effect sizes and importances of these sequence-level parameters to identify the most influential factors affecting trimming and ligation outcomes. The strongest positive effects were observed for trimming-related germline-encoded microhomology effects (parameter = 0.4484), followed by the presence of a C nucleotide immediately 5’ of the J-gene trimming site (parameter_*J*_ = 0.1308), and ligation-related germline-encoded microhomology effects (parameter = 0.1272) (Fig. 3A and Supplementary Fig. S9). In contrast, the most negative effects were an increase in GC nucleotides 3’ of the motif for both V- and J-genes (parameter_*V*_ = −0.1321, parameter_*J*_ = −0.1512), the presence of a C nucleotide 3’ of the V-gene trimming site (parameter_*V*_ = −0.1049), and an increase in AT nucleotides 5’ of the motif for both V- and J-genes (parameter_*V*_ = −0.1095, parameter_*J*_ = −0.1030). *P*-values corresponding to each of these effects were smaller than machine tolerance (*p* ≃ 0).

To evaluate the relative importance of model parameters, we trained our model using standardized features which ensure that parameter estimates directly reflect their relative importance to the model. This analysis revealed that the counts of AT and GC nucleotides 3’ of the motif for both V- and J-genes were the most influential, closely followed by the parameter representing trimming-related microhomology effects (Fig. 3C). Parameters corresponding to the counts of GC nucleotides 5’ of the motif and ligation-related microhomology effects were also identified as relatively important.

### Germline-encoded microhomology significantly improves model fit for predicting trimming and ligation across other receptor loci and sequence types

To further assess the importance of incorporating germline-encoded microhomology-related parameters for accurately predicting trimming and ligation probabilities, we compared the performance of a full model, which includes germline-encoded microhomology, motif, and 5’ and 3’ base count terms, to models lacking specific terms. All models were trained using the non-productive TCRα training dataset and the parameters were held constant for subsequent analyses.

**Figure 4.**
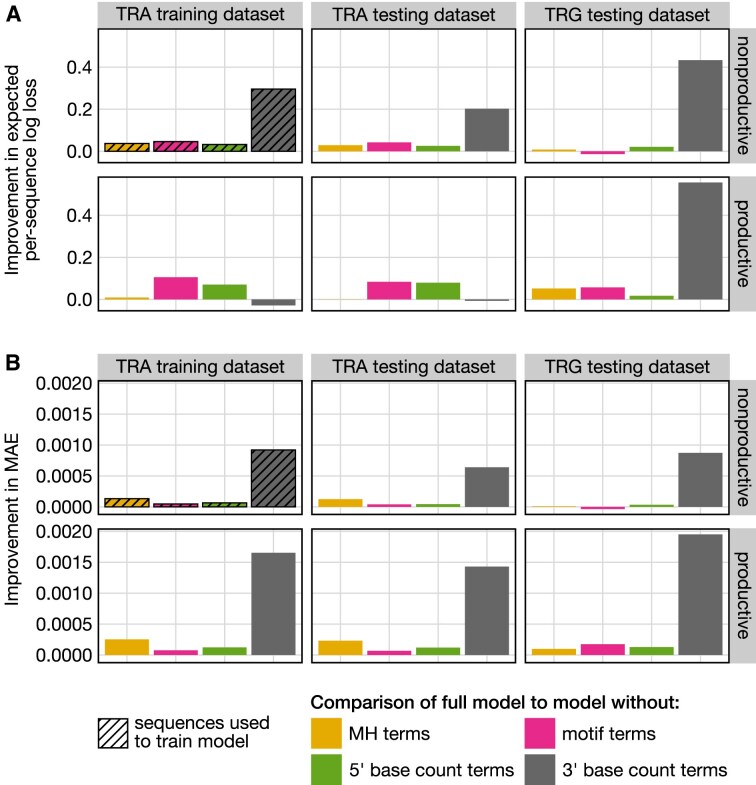
(**A**) Improvement in expected per-sequence log loss for the full model, which includes germline-encoded microhomology (MH) terms, motif terms, and 5’ and 3’ base count terms, compared to models without specific terms across both productive and non-productive sequences from multiple datasets. Improvement is the negative difference in log loss, with negative values indicating a relatively worse fit and positive values indicating a relatively better fit for the full model. Including MH terms improves log loss across all datasets, except for productive sequences from the TCRα testing dataset, where no change in loss was observed. (**B**) Improvement in MAE across the same models and datasets. Improvement is the negative difference in MAE, with negative values indicating relatively lower predictive accuracy and positive values indicating relatively higher predictive accuracy for the full model. Including MH terms consistently improves MAE across all datasets. All models were trained using non-productive sequences from the TCRα training dataset (hatched boxes), with parameters held constant (“frozen”) before calculating log loss and MAE across datasets.

We began by evaluating model performance on the training dataset. The full model showed a substantially lower expected per-sequence log loss compared to the model without germline-microhomology-related parameters, indicating a better fit to the data (Fig. 4A). This improvement was validated by a LRT, which confirmed the statistical significance of including germline-encoded microhomology terms (LRTstatistic = 93754.84; *P*-value less than machine tolerance, *p* ≃ 0). The full model also exhibited higher predictive accuracy, as indicated by a lower mean absolute error (MAE = 0.00468) compared to the model without germline-encoded microhomology terms (MAE = 0.00481). We repeated this analysis across models lacking other parameter types and found that the full model consistently outperformed them, exhibiting lower expected per-sequence log loss and MAE in each case (Fig. 4). Recall that the 5’ base count parameters capture potential sequence-breathing effects by reflecting preferences for GC content upstream of the motif, while the 3’ base count parameters capture both preferences for the absolute position of the trimming site and sequence-breathing effects related to AT and GC content downstream of the motif. Among the individual parameter types, the 3’ base count terms had the largest impact, leading to the greatest improvement in both log loss and MAE. Microhomology and motif terms contributed the second-largest improvements in MAE and log loss, respectively. That is, the absolute position of the trimming site, represented by the 3’ base count terms, had the strongest influence, while the local nucleotide context at the trimming site (captured by motif terms) and the extent of germline-encoded microhomology between the trimmed and ligated sequences also provided positive contributions, though to a lesser extent. Sequence-breathing capacity upstream of the trimming site, reflected by the 5’ base count terms, improved log loss and MAE as well, but had a smaller overall effect compared to the other parameters.

Using frozen coefficients from our models trained on non-productive TCRα sequences without N-insertions, we can also infer trimming and ligation probabilities for productive sequences or sequences from other receptor loci. However, because our models are specifically designed for sequences lacking N-insertions—since N-insertions complicate ligation pattern analysis due to their unknown nucleotide composition prior to ligation—its inferences are limited to such sequences. To evaluate model performance, we tested all models on both productive and non-productive sequences from independent TCRα and TCRγ datasets. The full model consistently demonstrated superior predictive accuracy, achieving lower expected per-sequence log loss and MAE compared to alternative models (Fig. 4).

In most datasets, the inclusion of 3’ base count terms continued to have the strongest impact on improving model fit and predictive accuracy. However, there were two notable exceptions in log loss calculations for productive sequences from the TCRα training and testing datasets. In these cases, including 3’ base count terms, which capture effects related to the absolute positioning of the trimming site, negatively affected log loss. Since productive sequences are subject to selection-related effects that may alter preferences for trimming site positioning, the 3’ base count terms learned from non-productive sequences—where these selection effects are absent—may be less effective for predicting trimming in productive sequences. Nevertheless, the inclusion of 3’ base count terms still improved MAE in these cases, despite the negative impact on log loss. This discrepancy may stem from log loss being more sensitive to outliers than MAE.

The inclusion of microhomology terms also improved model fit and predictive accuracy across most datasets, consistently providing the second-largest improvement in MAE. Notably, even when applied to productive sequences from the TCRα testing set—despite these sequences not being included in training and having skewed recombination statistics due to selection—the full model outperformed the model without microhomology terms in MAE, although the log loss values were similar. This suggests that while the inclusion of microhomology terms improves log loss across datasets, their most pronounced impact is on MAE. Overall, these consistent findings across other receptor loci (i.e. TCRγ) and sequence types (i.e. productive sequences) highlight the biological significance of germline-encoded microhomology in accurately modeling trimming and ligation scenarios.

### Accounting for germline-encoded microhomology affects sequence annotation

Given the significant role of germline-encoded microhomology in predicting trimming and ligation scenarios across TCRα and TCRγ receptor loci, we wanted to evaluate how germline-encoded microhomology parameterization influences sequence annotation. Recall that sequence annotation involves assigning a specific V(D)J recombination annotation, which describes the associated trimming, insertion, and ligation scenarios, to an observed sequence. In earlier sections, we examined the joint probabilities of trimming and ligation scenarios *for V-J gene pairs*, which represent the normalized probability of each trimming and ligation scenario within the complete set of possibilities for a given gene pair. Here, we shift our focus to V(D)J recombination annotation probabilities *for individual observed sequences*, which represent the normalized probability of each V(D)J recombination annotation within all possible annotations for a given sequence. Since we are analyzing sequences without N-insertions, each V(D)J recombination annotation corresponds directly to a trimming and ligation scenario, allowing us to use our inferred joint trimming and ligation scenario distributions to calculate the corresponding V(D)J recombination annotation probabilities. In this analysis, we compare the V(D)J recombination annotation probabilities and rankings between two models: (1) the full model, which includes germline-encoded microhomology, motif, and 5’ and 3’ base count terms, and (2) a version of the model that excludes germline-encoded microhomology terms.

As expected from our earlier results, accounting for germline-encoded microhomology effects substantially alters annotation probabilities and their rankings for sequences with multiple possible annotations. In total, 9.2% of all sequences lacking N-insertions have a different top-ranked annotation when using the model that parameterizes germline-encoded microhomology compared to the model that does not. These sequences represent the subset where microhomology-related effects could be inferred given our model setup and were actually detected.

However, our model can only detect germline-microhomology-related effects in sequences that allow for such inference. The majority of N-insertion-lacking sequences (76.1%) have only one possible annotation, meaning microhomology-related effects could not influence their ranking. Among sequences with multiple possible annotations—where microhomology-related effects could, in principle, be detected—38.3% exhibit a change in the top-ranked annotation.

Since sequences without N-insertions tend to have fewer possible annotations than those with N-insertions, the ability to detect microhomology-related annotation effects is more limited in this subset. If we were to quantify these effects in sequences containing N-insertions, we might expect a larger fraction of sequences to have a different top-ranked annotation when microhomology is accounted for.

**Figure 5.**
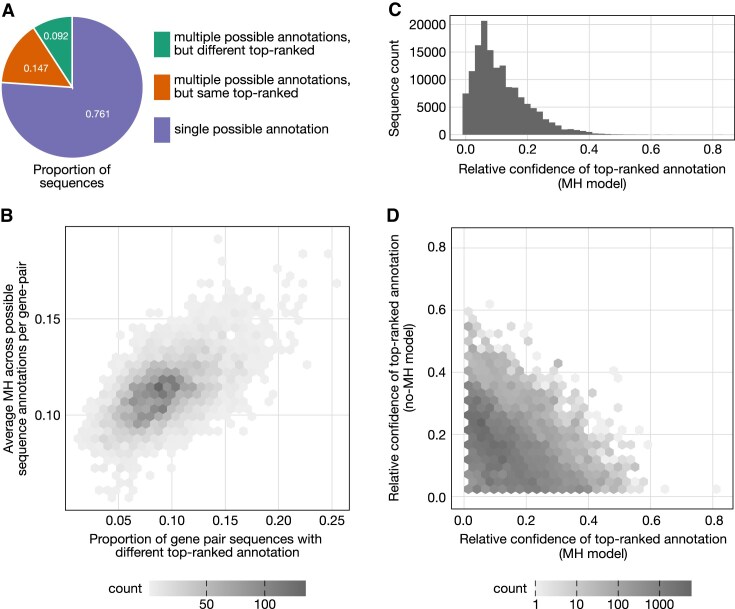
Accounting for germline-encoded microhomology (MH) in models used for sequence annotation substantially alters both V(D)J recombination annotation probabilities and rankings. For each sequence, the top-ranked annotation was determined separately using the model that parameterizes germline-encoded microhomology (MH model) and the model that does not (no-MH model). (**A**) Proportions of sequences categorized by whether they have single or multiple possible V(D)J recombination annotation scenarios and whether their top-ranked V(D)J recombination annotation differs between the MH and no-MH models. The majority of sequences lacking N-insertions have only one possible annotation, meaning microhomology-related effects could not influence their ranking. These statistics are specific to sequences without N-insertions, where the ability to detect microhomology-related annotation effects is more limited due to the generally lower number of possible annotations in this subset. If these effects were quantified in sequences containing N-insertions, a larger fraction would likely exhibit differences in top-ranked annotations when accounting for microhomology. (**B**) Correlation between the proportion of sequences with differing top-ranked annotations (between the two models) and the average germline-encoded microhomology across potential annotations per sequence for each V-J gene pair. This highlights how germline-encoded microhomology may influence ranking changes across V-J gene pairs. (**C**) Distribution of relative confidence for the top-ranked annotation using the MH model. Relative confidence is defined as the absolute difference in annotation probabilities using the MH model, comparing the top-ranked annotation from the MH model to the top-ranked annotation from the no-MH model for each sequence. (**D**) Comparison of relative annotation confidence for each sequence between the two models. Relative confidence for a model is calculated as the absolute difference in annotation probabilities (from that model) for the two top-ranked annotations identified by the MH and no-MH models. Most sequences exhibit substantial shifts in relative confidence between the two models, highlighting large model-driven changes in sequence annotations, even when one or both models show high relative confidence.

For sequences lacking N-insertions where microhomology does influence annotation rankings, the magnitude of this effect appears to depend on the amount of germline-encoded microhomology present. Specifically, as the average germline-encoded microhomology across potential annotations increases for a given sequence within a V-J gene pair, the proportion of gene pair sequences with differing top-ranked annotations between the two models also increases (Fig. 5B). We quantified the significance of this relationship using Pearson’s correlation, which revealed a moderately positive correlation (*r* = 0.5877; p-value smaller than machine tolerance, *p* ≃ 0). For some V-J gene pairs, parameterizing germline-encoded microhomology has a particularly pronounced impact on sequence annotation. For example, sequences involving TRAV38-1*04 and TRAJ22*01 show a striking difference in annotation predictions between models, with 25.13% of sequences exhibiting different top-ranked annotations. This effect may be driven by the relatively high germline-encoded microhomology content across annotations for these sequences, averaging 0.1697 nucleotides compared to the overall average of 0.1144 nucleotides across all V-J gene pairs.

Given that parameterizing germline-encoded microhomology leads to different top-ranked annotations for many sequences, we next quantified the relative confidence of these rankings. To explore this, we compared the annotation probabilities assigned by the model with germline-encoded microhomology terms for the top-ranked annotations from the microhomology model and the no-microhomology model for each sequence. We define the relative confidence of a top-ranked annotation as the absolute difference in annotation probabilities compared to the top-ranked annotation from the other model. On average, for sequences containing a different top-ranked annotation between the two models, we find that the relative confidence of the top-ranked annotation for the microhomology model is 0.1140 (Fig. 5 C). This means that, on average, the top-ranked annotation from the microhomology model is 11.4% more probable than the top-ranked annotation from the no-microhomology model, based on probabilities assigned by the microhomology model.

Additionally, we examined the relative confidence levels of the top-ranked annotations from both models. If germline-encoded microhomology merely resolved ties between competing annotations, we would expect minimal relative confidence in the top-ranked annotation using the model lacking microhomology terms, with larger relative confidence observed for the model containing microhomology terms. However, our findings indicate substantial shifts in relative confidence across models for most sequences (Fig. 5 D), with data points widely distributed rather than clustering near the axes. For instance, even when the model lacking microhomology terms has high confidence in its top-ranked annotation relative to the top-ranked annotation derived from the model containing microhomology, a similar flip in confidence is often observed when switching models. This effect suggests that parameterizing germline-encoded microhomology leads to meaningful changes in the annotation ranking landscape, potentially altering the biological interpretation of many sequences.

Beyond sequence annotation probabilities and rankings, we were interested in exploring whether germline-microhomology-related effects had practical implications for sequence generation probabilities, which are often used to characterize immune repertoire sequences. Our analysis revealed a small but consistent difference in sequence generation probabilities between the model that includes microhomology effects and the one that does not (Supplementary Fig. S10). Notably, these differences correlate with the average number of microhomologous nucleotides across all possible annotation scenarios for a given sequence (Supplementary Fig. S11), likely reflecting the influence of microhomology on sequence generation. These results suggest that incorporating microhomology effects into generative models of immune repertoire sequencing could enhance their biological relevance and improve their utility as negative controls, a common application in the literature [41, 47, 48].

## Discussion

Previous *in vitro* experiments have suggested that germline-encoded microhomology plays a significant role in biasing key V(D)J recombination processing steps, such as trimming and ligation. However, these findings do not fully elucidate the importance of germline-encoded microhomology in shaping *in vivo* recombination outcomes in humans. In this paper, we use statistical inference on previously-published high-throughput human TCR repertoire data [38, 39] to assess whether germline-encoded microhomology influences V(D)J recombination in humans with intact recombination machinery. Our probabilistic modeling framework quantifies how sequence-level features, particularly germline-encoded microhomology, impact trimming and ligation decisions during V(D)J recombination. We find that (1) germline-encoded microhomology significantly increases trimming and ligation event probabilities such that each additional nucleotide of microhomology increases the odds of a trimming event by 181% and the odds of a ligation event by 34%, (2) incorporating germline-encoded microhomology terms significantly enhances model fit for predicting trimming and ligation across multiple receptor loci and sequence types, and (3) accounting for microhomology when inferring V(D)J recombination annotations alters annotation probabilities and rankings, leading to a qualitatively different top-ranked annotation for 38.2% of sequences with multiple possible annotations.

Our results reveal that germline-encoded microhomologous nucleotides between gene ends significantly increase the probabilities of ligation events, aligning with previous *in vitro* evidence suggesting that germline-encoded microhomology guides ligation [3–9]. While much of the previous experimental focus has been on terminal microhomology (present at gene ends), many gene pairs lack terminal microhomology but have interior regions of microhomology. It has been proposed that trimming can expose these interior regions, which then guide ligation through germline-microhomology-mediated processes [7, 8]. Our findings support this, as germline-encoded microhomology appears to have a stronger effect on trimming than on ligation, likely due to the dependence of ligation options on prior trimming choices. Because this analysis focuses on sequences without N-insertions—allowing us to directly identify germline-microhomology-mediated ligation events—the observed strength of these effects may be amplified compared to analyses that include all sequences. Notably, when analyzing sequences with N-insertions—where ligation is not mediated by germline-encoded microhomology—we still observe that germline-encoded microhomology influences trimming, though less strongly, suggesting a more complex role for germline-encoded microhomology in V(D)J recombination beyond its involvement in ligation.

In addition to germline-microhomology-related parameters, our modeling framework included sequence-level parameters designed to capture the effects of local nucleotide context, absolute trimming site positioning, and sequence breathing capacity. These parameters, except for the germline-microhomology-related ones, were introduced in our previous analyses of trimming patterns for individual V- and J-gene sequences [26]. Our current results were consistent with those earlier findings. Specifically, parameters capturing the effects of absolute trimming site positioning and sequence breathing capacity downstream of the trimming site had the most substantial impact on improving overall model fit and predictive accuracy, showing the largest negative effect sizes on trimming and ligation choices. This pattern held when evaluating model performance and accuracy with sequences from different receptor loci and productivity types (e.g. TCRγ sequences and productive TCRα sequences), highlighting the influence of germline-encoded microhomology on recombination decisions. An important next step could involve investigating microhomology-related effects in other receptor loci that are more challenging to study, such as TCRβ, which has less sequence diversity between joining genes, and *IGH*, which undergoes post-recombination somatic hypermutation.

Beyond its intrinsic interest, germline-encoded microhomology has significant practical implications. In addition to influencing trimming and ligation probabilities, we found that parameterizing germline-microhomology-related effects leads to shifts in V(D)J recombination annotation probabilities and rankings, as well as sequence generation probabilities. These shifts often corresponded to large changes in the relative confidence of annotation rankings when comparing models that incorporate germline-encoded microhomology with those that do not. Such changes could meaningfully alter the annotation and sequence generation probability landscape, potentially impacting the biological interpretation of many sequences.

Our analysis was restricted to sequences lacking N-insertions, which tend to have fewer possible annotations per sequence compared to those with N-insertions. Because microhomology-related annotation effects can only be detected in sequences with multiple possible annotations, the ability to observe these effects is inherently more restricted in this subset. If we were to quantify these effects in sequences containing N-insertions, a larger fraction would likely exhibit differences in top-ranked annotations when microhomology is accounted for. Despite these findings, to our knowledge, all widely used V(D)J recombination annotation software [37, 49, 50] and generative models of immune repertoire sequencing data [37] do not account for germline-encoded microhomology or consider annotations that incorporate germline-encoded microhomologous nucleotides.

Our work has several limitations. First, we rely on non-productive rearrangements as a proxy for pre-selection recombination statistics, as is common in the literature [25, 37, 40, 41]. Non-productive sequences are sequenced as part of the repertoire when they coexist within a cell expressing a productive rearrangement that has passed the selection process. While we are not aware of any mechanism that could correlate non-productive and productive rearrangements within a single cell, nor of any evolutionary pressures acting to minimize the frequency of non-productive rearrangements, we acknowledge that the repertoire of non-productive rearrangements may not perfectly reflect the pre-selection repertoire. Nevertheless, we assume that recombination events are independent and that non-productive rearrangements reasonably approximate the recombination statistics of the repertoire before selection. Second, our analysis excluded sequences with N-insertions, allowing us to use known germline V- and J-gene sequences to identify regions of germline-encoded microhomology and potential germline-microhomology-mediated ligation events. N-insertions complicate the analysis because the identities of inserted nucleotides are unknown, and their presence suggests germline-encoded microhomology did not guide ligation. Because the presence or absence of N-insertions may affect the probability of successful ligation, excluding these sequences could shift the distribution of observed trimming and ligation events. Consequently, the germline microhomology effects that we have inferred may not extend to sequences with N-insertions. Future work could explore insertion-dependent microhomology dynamics in sequences containing N-insertions, but doing so would require assumptions about and integration over latent variables such as N-insertion identities prior to ligation, making this analysis challenging if using repertoire sequencing data. Relatedly, future work could also investigate how microhomology influences gene usage inference during V(D)J recombination annotation.

Despite the clear role of germline-encoded microhomology in biasing V(D)J recombination events and influencing V(D)J recombination annotation inference, no probabilistic models incorporating microhomology have been developed, to our knowledge. Future work could integrate microhomology-related dependencies into existing probabilistic frameworks like IGoR [37], which currently models dependencies between recombination events such as V- and J-gene choice, V-gene choice and V-gene deletions, and J-gene and J-gene deletions for TCRα sequences. To explicitly account for microhomology, additional dependencies would need to be introduced between V- and J-gene deletions, V-gene choice and J-gene deletion, and J-gene choice and V-gene deletion, along with incorporating new parameters to capture the sharing of germline-encoded microhomologous nucleotides. However, this approach could be challenging due to the large number of parameters required and the corresponding need for large datasets to adequately train the model. Alternatively, one could replace junctional processing event terms (such as those related to trimming and insertion) within IGoR with a more generalized model of junctional processing that incorporates microhomology, such as the model presented here. This modification would substantially reduce the number of required parameters, potentially balancing model complexity with practicality, although it might limit the ability to capture gene-specific processing profiles. Other more advanced approaches, such as combining simulation with deep learning, could also be explored to account for microhomology in V(D)J recombination annotation inference.

In summary, our findings demonstrate that germline-encoded microhomology plays a significant role in trimming and ligation choices during V(D)J recombination, underscoring the importance of accounting for germline-encoded microhomology effects when predicting recombination outcomes and annotating sequences. By advancing our understanding of the influence of germline-encoded microhomology in human V(D)J recombination, these results provide another step toward uncovering how this process generates diverse receptors that support a robust immune response in humans.

## Supplementary Material

gkaf250_Supplemental_File

## Data Availability

Code implementing the modeling described is available at https://github.com/magdalenarussell/microhomology and https://doi.org/10.6084/m9.figshare.27737685. The data used in this study were previously published and can be accessed through the Adaptive Biotechnologies immuneACCESS database via the links provided in the original publications [38, 39, 42].
