# Supplementary material for: Statistical analysis of repertoire data demonstrates the influence of microhomology in V(D)J recombination
Source: Nucleic Acids Res. 2025 Apr 2;53(6):gkaf250. doi: 10.1093/nar/gkaf250 (PMC11963759; doi:10.1093/nar/gkaf250)
Supplement: gkaf250_Supplemental_File [file gkaf250_supplemental_file.pdf]

Statistical analysis of repertoire data demonstrates the influence of microhomology in V(D)J recombination

Magdalena L Russell, Assya Trofimov, Philip Bradley, Frederick A Matsen IV

To whom correspondence should be addressed. E-mail: magruss@uw.edu or matsen@fredhutch.org

Contents

1 Supporting Information Text **1**

2 Supporting Figures and Tables **12**

3 SI References **25**

1 Supporting Information Text

TCRα training dataset

We downloaded TCRα repertoire sequence data from thymocyte samples of 10 immunologically healthy infants (aged 0-1 years) from the Adaptive Biotechnologies immuneACCESS database, following links provided in the original publications [5, 4].

Initial V(D)J recombination annotations were assigned to each sequence using the IGoR software (version 1.4.0) [10], which generates potential recombination annotations alongside their corresponding likelihoods. For each sequence, we identified the ten highest-probability recombination annotations and sampled one annotation based on posterior probabilities to assign an initial annotation. This included V- and J-gene assignments, trimming lengths, and the number of N-insertions.

Using these initial annotations, we filtered out sequences with inferred N-insertions (as determined by IGoR) to focus on potential germline microhomology-mediated ligation events. Sequences with N-insertions were excluded because their presence likely indicates that ligation did not involve germline microhomology. For the remaining sequences, we processed the initial annotations to determine all possible trimming and ligation scenarios based on the IGoR-inferred V- and J-gene assignments and N-insertion amounts. Since IGoR does not explicitly account for microhomology and assigns shared nucleotides to only one gene segment, we did not directly use the IGoR-inferred trimming annotations. Instead, we adapted the trimming scenario annotations to account for germline-encoded microhomology, generating a set of possible trimming and ligation scenarios for each sequence, including scenarios involving germline-encoded microhomologous nucleotides (see later Supplementary Materials for more details).

To finish preparing the training dataset, we excluded sequences that had more than fourteen nucleotides trimmed from either the V-gene or J-gene, as more extensive trimming is uncommon and could suggest an alternative trimming mechanism. We also focused only on non-productive sequences to avoid the potential confounding effects of selection in recombination statistics. After applying these filtering criteria, the final training dataset consisted of 1,257,528 sequences. To validate our model, we used a separate dataset of 983,514 productive sequences.

TCRα testing dataset

We downloaded TCRα repertoire sequence data from peripheral blood samples of 10 healthy individuals (aged 3-14 years) from the Adaptive Biotechnologies immuneACCESS database using the link in the original

publication [4]. This cohort differs from the training cohort in demographics and sampling location. All individuals were heterozygous for HLA-DR3/DR4 and had siblings diagnosed with Type-1 Diabetes, but they showed no clinical symptoms of diabetes at the time of sampling or in the subsequent years. We applied the same IGoR-based annotation and filtering procedures to this testing dataset as used for the training dataset. For model validation, we separately analyzed 98,244 non-productive and 141,676 productive sequences.

TCRγ testing dataset

Annotated TCRγ repertoire sequence data for 23 healthy bone marrow donor subjects was downloaded from the Adaptive Biotechnologies immuneACCESS database [12]. We applied the same filtering procedures to this testing dataset as used for the training dataset. For model validation, we separately analyzed approximately 44,673 non-productive and 20,681 productive sequences.

Identifying the set of possible trimming and ligation annotations for a sequence

We aim to identify all feasible combinations of trimming and ligation values (delV, delJ, and MH) that could account for the observed sequence X. Recall that V and J represent the V-gene and J-gene, which each define a V-gene and J-gene sequence, respectively. For ease of notation, these sequences are both oriented in the 3'-to-5' direction and are represented as ordered lists of nucleotides. Although the top strand of the V-gene typically follows a 5'-to-3' orientation, we reverse it here for notational convenience.

Recall that each sequence receives an initial annotation inferred by IGoR, consisting of a V-gene and J-gene assignment, trimming scenario, and N-insertion amount. While we use the gene and N-insertion assignments directly, we do not use the IGoR-inferred trimming scenario as-is. This is because IGoR does not explicitly account for microhomology and assigns shared nucleotides to only one gene segment. Instead, we adapt the trimming annotations to incorporate possible germline-encoded microhomology, generating a set of possible trimming and ligation scenarios for each sequence, including those involving microhomologous nucleotides.

To begin, let delV_i and delJ_i represent specific V- and J-gene trimming amounts inferred by IGoR in the initial annotation. Since IGoR assumes MH = 0, the initial set of possible annotations, A_X, includes only $(\text{delV} = \text{delV}_i, \text{delJ} = \text{delJ}_i, \text{MH} = 0)$. To expand A_X to include annotations with microhomology $(\text{MH} > 0)$, we introduce a function $h(x, y)$, which quantifies the number of contiguous complementary nucleotides between two overlapping, equal-length sequence regions x and y. This function is defined as:

$$h(x,y) = \sum_{i=0}^{\text{len}(x)} \begin{cases} 1 & \text{if } x(j) \text{ is complementary to } y(j) \text{ for all } j \in \{0,\dots,i\} \\ 0 & \text{otherwise.} \end{cases} \tag{1}$$

We apply this function to assess complementarity in overlapping regions between a V-gene sequence (V) and a J-gene sequence (J), aligning the sequences without gaps at the IGoR-inferred trimming sites delV_i and delJ_i (Figure S12). The overlapping regions are:

1. The *V-gene:trimmed-J* region, where $\texttt{seq}_{\texttt{trimmed}}(\text{J}, \text{delJ}_i)$ represents the trimmed J-gene sequence oriented 5'-to-3', and $\texttt{seq}_{\texttt{overlap}}(\text{V}, \text{delV}_i, \text{delJ}_i)$ represents the overlapping V-gene sequence oriented 3'-to-5'.

2. The *J-gene:trimmed-V* region, with $\texttt{seq}_{\texttt{trimmed}}(\text{V}, \text{delV}_i)$ and $\texttt{seq}_{\texttt{overlap}}(\text{J}, \text{delJ}_i, \text{delV}_i)$ representing the trimmed V-gene and overlapping J-gene sequences oriented 5'-to-3' and 3'-to-5', respectively.

We define k_J and k_V as the counts of contiguous complementary nucleotides in these regions:

$$k_J = h\big(\texttt{seq}_{\texttt{overlap}}(\text{V}, \text{delV}_i, \text{delJ}_i), \texttt{seq}_{\texttt{trimmed}}(\text{J}, \text{delJ}_i)\big)$$

and

$$k_V = h\big(\texttt{seq}_{\texttt{overlap}}(\text{J}, \text{delJ}_i, \text{delV}_i), \texttt{seq}_{\texttt{trimmed}}(\text{V}, \text{delV}_i)\big).$$

Given these values, a sequence X with an initial IGoR-inferred trimming scenario $(\text{delV} = \text{delV}_i, \text{delJ} = \text{delJ}_i, \text{MH} = 0)$ can also be annotated with microhomologous nucleotide counts MH ranging from 0 to

$k_V + k_J$. For each of these values of MH, the corresponding trimming amounts (delV and delJ) are adjusted accordingly to ensure the same observed sequence is generated. This expands the set of possible annotations A_X to:

$$A_X = \{(\text{delV}_i, \text{delJ}_i, 0)\} \cup \{(\text{delV}_n, \text{delJ}_n, m) \mid \text{conditions}\}.$$

The conditions are:

1. For each possible annotation, delV_n and delJ_n (realizations of delV and delJ) are within the range of initial IGoR-inferred trimming values adjusted by the contiguous nucleotide count: $\text{delV}_i - k_V \leq \text{delV}_n \leq \text{delV}_i$ and $\text{delJ}_i - k_J \leq \text{delJ}_n \leq \text{delJ}_i$.

2. The microhomologous nucleotide count m (a realization of MH) ranges from 0 to the sum of contiguous complementary nucleotides: $0 \leq m \leq k_V + k_J$.

3. The sum of the trimming amounts delV_n and delJ_n and the microhomologous nucleotide count m equals the sum of the initial IGoR-inferred trimming amounts: $\text{delV}_n + \text{delJ}_n + m = \text{delV}_i + \text{delJ}_i$

This process can be repeated to identify the sets of all possible sequence annotations for each sequence sampled from a TCRα repertoire.

Defining a model weight function

We aim to model the influence of various sequence-level parameters, including microhomology-related parameters, on joint trimming and ligation scenario probabilities, $P(\text{delVJ}, \text{MH} \mid \text{VJ}, \text{Q}, \text{I} = 0)$, where delVJ represents the trimming scenario (defined by delV and delJ), MH represents the number of microhomologous nucleotides used in ligation, VJ represents the gene pair, Q represents the productivity of the sequences, and I = 0 represents zero N-insertions. For our modeling purposes, we assume the following about V(D)J recombination biology:

1. The DNA hairpin of each joining gene is nicked open by a single-stranded break [3, 11, 9, 6, 8].

2. This hairpin nick occurs at the +2 position, creating a 4-nucleotide-long 3'-single-stranded overhang, with the two 3'-most nucleotides being P-nucleotides [9, 8].

3. If any part of the original gene sequence is deleted, all P-nucleotides will also be deleted [3, 14].

These assumptions allow us to determine the germline nucleotide sequence on both sides of each trimming site and define sequence-level model features. We assume that observations can be drawn from a model where these features vary across trimming and/or ligation scenarios for a given gene pair. Using these assumptions, we previously demonstrated that local nucleotide identity surrounding trimming sites (the "trimming motif") and the counts of GC or AT nucleotides beyond these motifs (the "5' base-count" and "3' base-count") are highly predictive of trimming probabilities for single gene sequences [13]. Building on this foundation, we aim to integrate these established parameters with newly developed microhomology-related parameters to assess the combined effects on the processes of trimming and ligation.

For our model, we define two sets of parameters, one trimming-related and one ligation-related, to model the probabilities of trimming and ligation scenarios. We model trimming scenario probabilities using established trimming motif (given by $\boldsymbol{\beta}_V^{\texttt{motif}}$ and $\boldsymbol{\beta}_J^{\texttt{motif}}$) and 5' and 3' base count (given by $\boldsymbol{\beta}_V^{\texttt{AT}}$, $\boldsymbol{\beta}_J^{\texttt{AT}}$, $\boldsymbol{\beta}_V^{\texttt{GC}}$, and $\boldsymbol{\beta}_J^{\texttt{GC}}$) parameters for each gene, along with a new parameter related to microhomology (given by $\beta^{\texttt{trimMH}}$). This new parameter measures the trimming-related effect of the average number of microhomologous nucleotides across all possible ligation scenarios for a given trimming scenario. Additionally, we model ligation scenario probabilities using another new parameter related to microhomology (given by $\beta^{\texttt{ligMH}}$), which measures the ligation-related effect of the number of microhomologous nucleotides within the ligation scenario. Using these model parameters, we define weight functions for the trimming choice $f_{\texttt{trim}}$ and the ligation choice $f_{\texttt{lig}}$ such that our model of $P(\text{delVJ}, \text{MH} \mid \text{VJ}, \text{Q}, \text{I} = 0)$ will be a normalized version of these weights.

The trimming-related weight function $f_{\texttt{trim}}$ aggregates the desired parameter-specific weight functions (defined in Table S2) as follows:

$$\begin{aligned} f_{\texttt{trim}}(\text{delVJ}, \text{VJ}; \boldsymbol{\beta}_{\texttt{trim}}) & \\ &:= f_{\texttt{motif}}(\text{delV}, \text{V}; \boldsymbol{\beta}_V^{\texttt{motif}}) + f_{\texttt{motif}}(\text{delJ}, \text{J}; \boldsymbol{\beta}_J^{\texttt{motif}}) \\ &+ f_{\texttt{count}}(\text{delV}, \text{V}; \boldsymbol{\beta}_V^{\texttt{AT}}, \boldsymbol{\beta}_V^{\texttt{GC}}) + f_{\texttt{count}}(\text{delJ}, \text{J}; \boldsymbol{\beta}_J^{\texttt{AT}}, \boldsymbol{\beta}_J^{\texttt{GC}}) \\ &+ f_{\texttt{trimMH}}(\text{delVJ}, \text{VJ}; \beta^{\texttt{trimMH}}). \end{aligned} \tag{2}$$

For notational convenience, $\boldsymbol{\beta}_{\texttt{trim}}$ represents the set of all trimming-related regression parameters, $\text{VJ} = (\text{V}, \text{J})$ represents a gene pair, $\text{delVJ} = (\text{delV}, \text{delJ})$ represents a trimming scenario, and MH represents the number of microhomologous nucleotides within the ligation scenario.

Additionally, we define a ligation-related weight function $f_{\texttt{lig}}$ that consists of the relevant parameter-specific weight function (defined in Table S2) as follows:

$$f_{\texttt{lig}}(\text{delVJ}, \text{MH}, \text{VJ}; \boldsymbol{\beta}_{\texttt{lig}}) := f_{\texttt{ligMH}}(\text{MH}; \beta^{\texttt{ligMH}}) \tag{3}$$

where $\boldsymbol{\beta}_{\texttt{lig}}$ represents the set of all ligation-related regression parameters, which for our purposes includes only $\beta^{\texttt{ligMH}}$.

We further define each of these trimming-related and ligation-related regression parameters, along with their corresponding weight functions, within the following sections:

Defining "trimming motif" parameters

As in our previous work [13], we define trimming motif parameters to include one nucleotide position 5' of the trimming site and two nucleotide positions 3' of the trimming site. We describe this definition for a V-gene sequence and V-gene trimming amount, but it applies similarly to a J-gene sequence and J-gene trimming amount.

Recall that V represents the V-gene which defines a V-gene sequence. For ease of notation, we orient this sequence in the 3'-to-5' direction and represent it as an ordered list of nucleotides. Recall that delV is a random variable representing a V-gene trimming amount. Let $\text{V}(\text{delV} + 2 - j)$ represent the nucleotide identity at the trimming motif position $j \in \{0, \ldots, 2\}$ where positions $j \leq 0$ represent motif positions 5' of the trimming site and positions $j > 0$ represent motif positions 3' of the trimming site. The trimming motif sequence (oriented 5'-to-3') is given by the ordered list:

$$\big(\text{V}(\text{delV} + 2 - j)\big)_{j=0}^{2}. \tag{4}$$

Depending on delV, this trimming motif may or may not include P-nucleotides. For $\text{delV} \geq 2$, the two 3' trimming motif nucleotides will include the two deleted gene sequence nucleotides 3' of the trimming site (and no P-nucleotides). Since we are assuming that the initial hairpin nick occurs at the +2 position, there will be two P-nucleotides present in the 5'-to-3' gene sequence. For $0 \leq \text{delV} < 2$, P-nucleotides will be included in the trimming motif sequence. Likewise, as a result of the +2 hairpin nick position assumption, TCRs that have $\text{delV} < 0$ will not have a full-length nucleotide trimming motif. For these "off-the-end" motif cases, we assign zero influence to the missing nucleotides during model fitting.

Let $\beta_{jk}^{\texttt{motif}}$ be a (log) position-weight-matrix parameter for trimming motif position $j \in \{0, \ldots, 2\}$ and nucleotide $k \in \{A, T, C, G\}$. The set of all such parameters for the V-gene is denoted by $\boldsymbol{\beta}_V^{\texttt{motif}}$. We can define an un-normalized position-weight-matrix weight:

$$f_{\texttt{motif}}(\text{delV}, \text{V}; \boldsymbol{\beta}_V^{\texttt{motif}}) := \sum_{j=0}^{2} \beta_{j\text{V}(\text{delV}+2-j)}^{\texttt{motif}} \tag{5}$$

that will serve as a *motif*-specific weight function in subsequent modeling. As described above, since we are considering "off-the-end" motif cases, $\text{V}(\text{delV} + 2 - j)$ will represent the nucleotide identity at sequence position j where positions $j \leq 0$ represent sequence positions 5' of the trimming site and positions $j > 0$ represent sequence positions 3' of the trimming site.

Defining "base count" parameters

As in our previous work [13], we will also define parameters for the counts of GC and AT nucleotides on either side of each trimming site. We describe this definition for a V-gene sequence and V-gene trimming amount, but it applies similarly to a J-gene sequence and J-gene trimming amount. For an arbitrary sequence x, we can count the number of AT and GC nucleotides within the sequence as

$$C^{\texttt{AT}}(x) = C^{\texttt{A}}(x) + C^{\texttt{T}}(x) \tag{6}$$

and

$$C^{\texttt{GC}}(x) = C^{\texttt{G}}(x) + C^{\texttt{C}}(x), \tag{7}$$

respectively.

Since the count of AT or GC nucleotides within the sequences 5' and 3' of the trimming site may influence the probability of trimming differently, we calculate the counts separately and exclude nucleotides already included in the *motif* parameterization. As above, recall that delV represents a V-gene trimming amount and V represents a V-gene which defines a V-gene sequence. For ease of notation, we orient this sequence in the 3'-to-5' direction and represent it as an ordered list of nucleotides. Let $\mathrm{V}(\mathrm{delV} + 2 - j)$ represent the nucleotide identity at the trimming motif position $j \in \{0, \ldots, 2\}$ where positions $j \leq 0$ represent motif positions 5' of the trimming site and positions $j > 0$ represent motif positions 3' of the trimming site. As in our previous work, we include the ten nucleotides 5' of the motif in the 5' nucleotide counts. Since we include one nucleotide 5' of the trimming site in the "trimming motif" parameters (as described in the previous section), the nucleotide sequence 5' of the trimming site, beyond the "trimming motif", is given by the ordered list

$$\mathsf{seq}_5(\mathrm{delV}, \mathrm{V}) = \big(\mathrm{V}(\mathrm{delV} + 2 - j)\big)_{j=-11}^{-1}. \tag{8}$$

To count the number of AT and GC nucleotides in the sequence 3' of the trimming site, we include all nucleotides located 3' of the trimming site beyond the "trimming motif." Since we are interested in using GC nucleotide content as a proxy for sequence-breathing, which is relevant only for paired nucleotides, we exclude nucleotides within the 3' single-stranded overhang. Assuming the initial hairpin nick occurs at the +2 position, leading to a 4-nucleotide-long 3' single-stranded overhang, for delV > 2, the nucleotide sequence 3' of the trimming site, beyond the "trimming motif" (which contains two nucleotide positions 3' of the trimming site), is given by the ordered list:

$$\mathsf{seq}_3(\mathrm{delV}, \mathrm{V}) = \begin{cases} \big(\mathrm{V}(\mathrm{delV} + 2 - j)\big)_{j=3}^{(\mathrm{delV}-2)} & \text{if } (\mathrm{delV} - 2) \geq 3 \\ () & \text{if } (\mathrm{delV} - 2) < 3. \end{cases} \tag{9}$$

For $(\mathrm{delV} - 2) < 3$, all nucleotides 3' of the trimming site are considered single-stranded, and thus no nucleotides will be included in the sequence used to calculate the AT and GC base-counts.

With these sequences 5' and 3' of the trimming site, we define $\beta_{5V}^{\texttt{AT}}$, $\beta_{3V}^{\texttt{AT}}$, $\beta_{5V}^{\texttt{GC}}$, and $\beta_{3V}^{\texttt{GC}}$ to be V-gene specific *base count* model parameters for 5' and 3' sequence base-counts of AT and GC beyond the "trimming motif", respectively. The set of all such parameters for the V-gene are denoted by $\boldsymbol{\beta}_V^{\texttt{AT}}$ and $\boldsymbol{\beta}_V^{\texttt{GC}}$. With these parameters, we define a *base count* weight function:

$$\begin{aligned} f_{\texttt{count}}(\mathrm{delV}, \mathrm{V}; \boldsymbol{\beta}_V^{\texttt{AT}}, \boldsymbol{\beta}_V^{\texttt{GC}}) := \beta_{5V}^{\texttt{AT}} \cdot C^{\texttt{AT}}(\mathsf{seq}_5(\mathrm{delV}, \mathrm{V})) + \beta_{3V}^{\texttt{AT}} \cdot C^{\texttt{AT}}(\mathsf{seq}_3(\mathrm{delV}, \mathrm{V})) \\ + \beta_{5V}^{\texttt{GC}} \cdot C^{\texttt{GC}}(\mathsf{seq}_5(\mathrm{delV}, \mathrm{V})) + \beta_{3V}^{\texttt{GC}} \cdot C^{\texttt{GC}}(\mathsf{seq}_3(\mathrm{delV}, \mathrm{V})). \end{aligned} \tag{10}$$

using the functions $C^{\texttt{AT}}$ and $C^{\texttt{GC}}$ as defined in (6) and (7), respectively. As defined, these GC and AT base-counts for the 3' sequence are dependent on sequence length and provide a parameterization of both GC nucleotide content and length.

Defining "microhomology" parameters for trimming scenario choice

We can parameterize the average number of microhomologous nucleotides across possible ligation scenario choices for a given trimming scenario and define $\beta^{\texttt{trimMH}}$ to be an *microhomology* model parameter specific to trimming choice. We define a function g that returns this average value as follows:

$$g(\mathrm{delVJ}, \mathrm{VJ}) := \frac{\sum_{\mathrm{MH}' \in \mathcal{M}_{\mathrm{VJ},\mathrm{delVJ}}} \mathrm{MH}'}{|\mathcal{M}_{\mathrm{VJ},\mathrm{delVJ}}|}$$

such that $\mathcal{M}_{\mathrm{VJ,delVJ}}$ is the set of all possible ligation scenarios for the chosen trimming scenario delVJ and gene pair VJ. With this function, we can define a *microhomology* weight function

$$f_{\texttt{trimMH}}(\mathrm{delVJ}, \mathrm{VJ}; \beta^{\texttt{trimMH}}) := \beta^{\texttt{trimMH}} \cdot g(\mathrm{delVJ}, \mathrm{VJ}). \tag{11}$$

Defining "microhomology" parameters for ligation scenario choice

We can directly use MH, which represents the number of microhomologous nucleotides in an observed ligation scenario, as a parameter. We then define $\beta^{\texttt{ligMH}}$ as the *microhomology* model parameter for predicting the choice of ligation scenario. We use the term "observed" for this microhomology parameter because these particular microhomologous nucleotides directly participate in the ligation process and are homologous in the final sequence. As such, we can define a *microhomology* weight function:

$$f_{\texttt{ligMH}}(\mathrm{MH}; \beta^{\texttt{ligMH}}) := \beta^{\texttt{ligMH}} \cdot \mathrm{MH}. \tag{12}$$

Extended model formulation and training description

We aim to model the influence of various sequence-level parameters, including microhomology-related parameters, on joint trimming and ligation scenario probabilities, $P(\mathrm{delVJ}, \mathrm{MH} \mid \mathrm{VJ}, \mathrm{Q}, \mathrm{I} = 0)$, where delVJ represents the trimming scenario, MH represents the number of microhomologous nucleotides used in ligation, VJ represents the gene pair, Q represents the productivity of the sequences, and $\mathrm{I} = 0$ represents zero N-insertions. Modeling this probability is complex because the true trimming and ligation annotation of each sampled sequence is a latent variable that will depend on the model parameters. As described earlier, we obtain the set of possible trimming and ligation scenario annotations (delVJ and MH), denoted as A_X, for a given sequence X by transforming the initial IGoR-inferred annotation. We assign probabilities (or weights) to each potential annotation, and since these probabilities depend on the model parameters, we use an expectation-maximization algorithm for parameter inference. Below, we provide a detailed description of these steps.

We employ a two-step conditional logit model to capture the decision-making involved in selecting trimming and ligation scenarios for V-J gene pairs. Our model describes a generative process in two steps:

1. We model the probability, $P(\mathrm{delVJ} \mid \mathrm{VJ}, \mathrm{Q}, \mathrm{I} = 0)$, of choosing a trimming scenario delVJ for a given V-J gene pair VJ, sequence productivity Q, and N-insertion amount $\mathrm{I} = 0$. This probability is modeled by parameters specific to trimming scenarios.

2. We model the probability, $P(\mathrm{MH} \mid \mathrm{delVJ}, \mathrm{VJ}, \mathrm{Q}, \mathrm{I} = 0)$, of choosing a ligation scenario MH for a given trimming scenario delVJ, V-J gene pair VJ, sequence productivity Q, and N-insertion amount $\mathrm{I} = 0$. This probability is modeled by parameters specific to each ligation scenario.

The joint probability of a trimming scenario delVJ and a ligation scenario MH for a given V-J gene pair VJ, sequence productivity Q, and N-insertion amount $\mathrm{I} = 0$ can be factored as:

$$P(\mathrm{delVJ}, \mathrm{MH} \mid \mathrm{VJ}, \mathrm{Q}, \mathrm{I} = 0) = P(\mathrm{delVJ} \mid \mathrm{VJ}, \mathrm{Q}, \mathrm{I} = 0) \times P(\mathrm{MH} \mid \mathrm{delVJ}, \mathrm{VJ}, \mathrm{Q}, \mathrm{I} = 0).$$

Figure 2 depicts the two-step structure of our model, illustrating the decision-making process for an example V-J gene pair.

To incorporate characteristics of each possible trimming and ligation scenario in our model, we define parameter-specific weight functions such that our model of $P(\mathrm{delVJ}, \mathrm{MH} \mid \mathrm{VJ}, \mathrm{Q}, \mathrm{I} = 0)$ will be a normalized version of these weights. In our previous work, we established that local nucleotide identities at trimming sites (the "trimming motif") and the counts of GC or AT nucleotides beyond these motifs (the "5' base-count" and "3' base-count") are strong predictors of trimming probabilities for single gene sequences [13]. Building on this foundation, we have integrated these established parameters with newly developed microhomology-related parameters to assess the combined effects on the processes of trimming and ligation. First, we define the trimming-related weight function $f_{\texttt{trim}}(\mathrm{delVJ}, \mathrm{VJ}; \boldsymbol{\beta}_{\texttt{trim}})$ parameterized by as set of trimming-related parameters $\boldsymbol{\beta}_{\texttt{trim}}$, which includes previously established trimming motif and base count parameters, and a new trimming-related microhomology parameter. This new parameter measures the effect of the

average number of microhomologous nucleotides between two sequences, a value that varies depending on the chosen trimming scenario. Similarly, we define the ligation-related weight function $f_{\texttt{lig}}(\text{delVJ}, \text{MH}, \text{VJ}; \boldsymbol{\beta}_{\texttt{lig}})$ parameterized by a new ligation-related microhomology parameter $\boldsymbol{\beta}_{\texttt{lig}}$ which measures the effect of the number of microhomologous nucleotides between two sequences, a value that varies depending on the chosen trimming and ligation scenario. These parameters and weight functions are summarized in Table S2 and defined in detail in previous Supplementary Materials sections.

With these weight functions, our model estimates the joint probability of a trimming and ligation scenario (given by delVJ and MH) for a given V-J gene pair VJ, sequence productivity Q, and N-insertion amount $I = 0$, combining influences of regression parameters $\boldsymbol{\beta}_{\texttt{trim}}$ and $\boldsymbol{\beta}_{\texttt{lig}}$:

$$\begin{aligned} P(\text{delVJ}, \text{MH} \mid \text{VJ}, \text{Q}, I = 0; \boldsymbol{\beta}_{\texttt{trim}}, \boldsymbol{\beta}_{\texttt{lig}}) &:= \\ & P(\text{delVJ} \mid \text{VJ}, \text{Q}, \text{I} = 0; \boldsymbol{\beta}_{\texttt{trim}}, \boldsymbol{\beta}_{\texttt{lig}}) \times P(\text{MH} \mid \text{delVJ}, \text{VJ}, \text{Q}, \text{I} = 0; \boldsymbol{\beta}_{\texttt{lig}}). \end{aligned} \tag{13}$$

To model the trimming scenario probability $P(\text{delVJ} \mid \text{VJ}, \text{Q}, \text{I} = 0; \boldsymbol{\beta}_{\texttt{trim}}, \boldsymbol{\beta}_{\texttt{lig}})$, we expand conditional probability, giving:

$$P(\text{delVJ}, \text{VJ}, \text{Q}, \text{I} = 0; \boldsymbol{\beta}_{\texttt{trim}}, \boldsymbol{\beta}_{\texttt{lig}}) = P(\text{Q}, \text{I} = 0 \mid \text{delVJ}, \text{VJ}; \boldsymbol{\beta}_{\texttt{lig}}) \times P(\text{delVJ}, \text{VJ}; \boldsymbol{\beta}_{\texttt{trim}}).$$

This probability $P(\text{delVJ} \mid \text{VJ}, \text{Q}, \text{I} = 0)$ is parameterized by both trimming- and ligation-related parameters ($\boldsymbol{\beta}_{\texttt{trim}}$ and $\boldsymbol{\beta}_{\texttt{lig}}$) because the model is conditioned on sequence productivity (Q), which is jointly determined by trimming and ligation. This dependency ensures that trimming probabilities properly account for how productivity constraints prune the space of possible ligation scenarios associated with each trimming scenario, correcting for any biases introduced by this non-uniform pruning. As such, we model $P(\text{delVJ} \mid \text{VJ}, \text{Q}, \text{I} = 0; \boldsymbol{\beta}_{\texttt{trim}}, \boldsymbol{\beta}_{\texttt{lig}})$ as:

$$\begin{aligned} P(\text{delVJ} \mid \text{VJ}, \text{Q}, \text{I} = 0; \boldsymbol{\beta}_{\texttt{trim}}, \boldsymbol{\beta}_{\texttt{lig}}) &= \frac{P(\text{delVJ}, \text{VJ}, \text{Q}, \text{I} = 0; \boldsymbol{\beta}_{\texttt{trim}}, \boldsymbol{\beta}_{\texttt{lig}})}{\sum_{\text{delVJ}' \in \mathcal{D}} P(\text{delVJ}', \text{VJ}, \text{Q}, \text{I} = 0; \boldsymbol{\beta}_{\texttt{trim}}, \boldsymbol{\beta}_{\texttt{lig}})} \\ &= \frac{P(\text{Q}, \text{I} = 0 \mid \text{delVJ}, \text{VJ}; \boldsymbol{\beta}_{\texttt{lig}}) \cdot P(\text{delVJ}, \text{VJ}; \boldsymbol{\beta}_{\texttt{trim}})}{\sum_{\text{delVJ}' \in \mathcal{D}} P(\text{Q}, \text{I} = 0 \mid \text{delVJ}', \text{VJ}; \boldsymbol{\beta}_{\texttt{lig}}) \cdot P(\text{delVJ}', \text{VJ}; \boldsymbol{\beta}_{\texttt{trim}})} \\ &:= \frac{P(\text{Q}, \text{I} = 0 \mid \text{delVJ}, \text{VJ}; \boldsymbol{\beta}_{\texttt{lig}}) \cdot \exp\left(f_{\texttt{trim}}(\text{delVJ}, \text{VJ}; \boldsymbol{\beta}_{\texttt{trim}})\right)}{\sum_{\text{delVJ}' \in \mathcal{D}} P(\text{Q}, \text{I} = 0 \mid \text{delVJ}', \text{VJ}; \boldsymbol{\beta}_{\texttt{lig}}) \cdot \exp\left(f_{\texttt{trim}}(\text{delVJ}', \text{VJ}; \boldsymbol{\beta}_{\texttt{trim}})\right)} \end{aligned} \tag{14}$$

where $f_{\texttt{trim}}$ is the trimming-related weight defined in (2) and $\mathcal{D}$ is the set of all possible trimming scenarios for the specified sequence productivity Q and N-insertion amount I = 0. We model $P(\text{Q}, \text{I} = 0 \mid \text{delVJ}, \text{VJ}; \boldsymbol{\beta}_{\texttt{lig}})$ as:

$$\begin{aligned} P(\text{Q}, \text{I} = 0 | \text{delVJ}, \text{VJ}; \boldsymbol{\beta}_{\texttt{lig}}) = \frac{P(\text{delVJ}, \text{VJ}, \text{Q}, \text{I} = 0; \boldsymbol{\beta}_{\texttt{lig}})}{P(\text{delVJ}, \text{VJ}; \boldsymbol{\beta}_{\texttt{lig}})} &= \frac{\sum_{\text{MH}_1 \in \mathcal{M}_1} P(\text{MH}_1, \text{delVJ}, \text{VJ}, \text{Q}, \text{I} = 0; \boldsymbol{\beta}_{\texttt{lig}})}{\sum_{\text{MH}_2 \in \mathcal{M}_2} P(\text{MH}_2, \text{delVJ}, \text{VJ}; \boldsymbol{\beta}_{\texttt{lig}})} \\ &= \frac{\sum_{\text{MH}_1 \in \mathcal{M}_1} P(\text{MH}_1, \text{delVJ}, \text{VJ}; \boldsymbol{\beta}_{\texttt{lig}})}{\sum_{\text{MH}_2 \in \mathcal{M}_2} P(\text{MH}_2, \text{delVJ}, \text{VJ}; \boldsymbol{\beta}_{\texttt{lig}})} \\ &:= \frac{\sum_{\text{MH}_1 \in \mathcal{M}_1} \exp\left(f_{\texttt{lig}}(\text{delVJ}, \text{MH}_1, \text{VJ}; \boldsymbol{\beta}_{\texttt{lig}})\right)}{\sum_{\text{MH}_2 \in \mathcal{M}_2} \exp\left(f_{\texttt{lig}}(\text{delVJ}, \text{MH}_2, \text{VJ}; \boldsymbol{\beta}_{\texttt{lig}})\right)} \end{aligned} \tag{15}$$

given that $P(\text{MH}, \text{delVJ}, \text{VJ}, \text{Q}, \text{I} = 0; \boldsymbol{\beta}_{\texttt{lig}}) = P(\text{MH}, \text{delVJ}, \text{VJ}; \boldsymbol{\beta}_{\texttt{lig}})$. Here, $f_{\texttt{lig}}$ is the ligation-related weight defined in (12), $\mathcal{M}_1$ is the set of all possible ligation scenarios for the chosen trimming scenario delVJ, sequence productivity Q, and N-insertion amount I = 0 and $\mathcal{M}_2$ is the set of all possible ligation scenarios for the chosen trimming scenario delVJ.

Similarly, we model the ligation scenario probability $P(\text{MH} \mid \text{delVJ}, \text{VJ}, \text{Q}, \text{I} = 0; \boldsymbol{\beta}_{\texttt{lig}})$ as:

$$
\begin{aligned}
P(\text{MH} \mid \text{delVJ}, \text{VJ}, \text{Q}, \text{I} = 0; \boldsymbol{\beta}_{\texttt{lig}}) &= \frac{P(\text{MH}, \text{delVJ}, \text{VJ}, \text{Q}, \text{I} = 0; \boldsymbol{\beta}_{\texttt{lig}})}{\sum_{\text{MH}_1 \in \mathcal{M}_1} P(\text{MH}_1, \text{delVJ}, \text{VJ}, \text{Q}, \text{I} = 0; \boldsymbol{\beta}_{\texttt{lig}})} \\
&= \frac{P(\text{MH}, \text{delVJ}, \text{VJ}; \boldsymbol{\beta}_{\texttt{lig}})}{\sum_{\text{MH}_1 \in \mathcal{M}_1} P(\text{MH}_1, \text{delVJ}, \text{VJ}; \boldsymbol{\beta}_{\texttt{lig}})} \\
&:= \frac{\exp\left(f_{\texttt{lig}}(\text{delVJ}, \text{MH}, \text{VJ}; \boldsymbol{\beta}_{\texttt{lig}})\right)}{\sum_{\text{MH}_1 \in \mathcal{M}_1} \exp\left(f_{\texttt{lig}}(\text{delVJ}, \text{MH}_1, \text{VJ}; \boldsymbol{\beta}_{\texttt{lig}})\right)}.
\end{aligned} \tag{16}
$$

Combining these, our model becomes

$$
\begin{aligned}
&P(\text{delVJ}, \text{MH} \mid \text{VJ}, \text{Q}, \text{I} = 0; \boldsymbol{\beta}_{\texttt{lig}}, \boldsymbol{\beta}_{\texttt{trim}}) \\
&\quad := P(\text{delVJ} \mid \text{VJ}, \text{Q}, \text{I} = 0; \boldsymbol{\beta}_{\texttt{trim}}, \boldsymbol{\beta}_{\texttt{lig}}) \times P(\text{MH} \mid \text{delVJ}, \text{VJ}, \text{Q}, \text{I} = 0; \boldsymbol{\beta}_{\texttt{lig}}) \\
&\quad := \frac{P(\text{Q}, \text{I} = 0 \mid \text{delVJ}, \text{VJ}; \boldsymbol{\beta}_{\texttt{lig}}) \cdot \exp\left(f_{\texttt{trim}}(\text{delVJ}, \text{VJ}; \boldsymbol{\beta}_{\texttt{trim}})\right)}{\sum_{\text{delVJ}' \in \mathcal{D}} P(\text{Q}, \text{I} = 0 \mid \text{delVJ}', \text{VJ}; \boldsymbol{\beta}_{\texttt{lig}}) \cdot \exp\left(f_{\texttt{trim}}(\text{delVJ}', \text{VJ}; \boldsymbol{\beta}_{\texttt{trim}})\right)} \\
&\qquad \times \frac{\exp\left(f_{\texttt{lig}}(\text{delVJ}, \text{MH}, \text{VJ}; \boldsymbol{\beta}_{\texttt{lig}})\right)}{\sum_{\text{MH}_1 \in \mathcal{M}_1} \exp\left(f_{\texttt{lig}}(\text{delVJ}, \text{MH}_1, \text{VJ}; \boldsymbol{\beta}_{\texttt{lig}})\right)}.
\end{aligned} \tag{17}
$$

where $P(\text{Q}, \text{I} = 0 \mid \text{delVJ}, \text{VJ}; \boldsymbol{\beta}_{\texttt{lig}})$ is defined in (15).

Recall that our data consists of sequences and we are considering sets of all possible sequence annotations A_X for each sampled sequence X. Each annotation includes a trimming scenario delVJ and ligation scenario MH. To infer the parameters of our model $P(\text{delVJ}, \text{MH} \mid \text{VJ}, \text{Q}, \text{I} = 0; \boldsymbol{\beta}_{\texttt{lig}}, \boldsymbol{\beta}_{\texttt{trim}})$, defined in 17, while marginalizing over all possible sequence annotations for each sequence, we employ an expectation-maximization (EM) approach. This iterative algorithm proceeds as follows: starting with initial model parameters $\boldsymbol{\beta}_{\texttt{lig}}$ and $\boldsymbol{\beta}_{\texttt{trim}}$, we aim to update to improved parameters $\boldsymbol{\beta}'_{\texttt{lig}}$ and $\boldsymbol{\beta}'_{\texttt{trim}}$. We define the normalized conditional probability of a specific sequence annotation $(\text{delVJ} = \text{delVJ}_1, \text{MH} = \text{MH}_1) \in A_X$ given a sequence X with gene pair $\text{VJ} = \text{VJ}_1$ as:

$$
\begin{aligned}
&P_{\text{annot}}(\text{delVJ} = \text{delVJ}_1, \text{MH} = \text{MH}_1 \mid \text{VJ} = \text{VJ}_1, \text{Q} = \text{Q}_1, \text{I} = 0; X, \boldsymbol{\beta}_{\texttt{lig}}, \boldsymbol{\beta}_{\texttt{trim}}) \\
&\qquad = \frac{P(\text{delVJ}_1, \text{MH}_1 \mid \text{VJ}_1, \text{Q}_1, I = 0; \boldsymbol{\beta}_{\texttt{lig}}, \boldsymbol{\beta}_{\texttt{trim}})}{\sum_{(\text{delVJ}_n, \text{MH}_n) \in A_X} P(\text{delVJ}_n, \text{MH}_n \mid \text{VJ}_1, \text{Q}_1, I = 0; \boldsymbol{\beta}_{\texttt{lig}}, \boldsymbol{\beta}_{\texttt{trim}})}.
\end{aligned} \tag{18}
$$

Here, $P(\text{delVJ}_1, \text{MH}_1 \mid \text{VJ}_1, \text{Q}_1, I = 0; \boldsymbol{\beta}_{\texttt{lig}}, \boldsymbol{\beta}_{\texttt{trim}})$ is computed according to (17). With this, we then define the expected log-likelihood of new parameter estimates $\boldsymbol{\beta}'_{\texttt{lig}}$ and $\boldsymbol{\beta}'_{\texttt{trim}}$ given the current estimates $\boldsymbol{\beta}_{\texttt{lig}}$ and $\boldsymbol{\beta}_{\texttt{trim}}$ for a single sampled sequence X as:

$$
\begin{aligned}
&\ell(\boldsymbol{\beta}'_{\texttt{lig}}, \boldsymbol{\beta}'_{\texttt{trim}} \mid \boldsymbol{\beta}_{\texttt{lig}}, \boldsymbol{\beta}_{\texttt{trim}}; X, \text{Q}, \text{I} = 0) \\
&\quad = \sum_{(\text{delVJ}_n, \text{MH}_n) \in A_X} P_{\text{annot}}(\text{delVJ} = \text{delVJ}_n, \text{MH} = \text{MH}_n \mid \text{VJ} = \text{VJ}_n, \text{Q}, \text{I} = 0; X, \boldsymbol{\beta}_{\texttt{lig}}, \boldsymbol{\beta}_{\texttt{trim}}) \\
&\qquad \times \log P(\text{delVJ} = \text{delVJ}_n, \text{MH} = \text{MH}_n \mid \text{VJ} = \text{VJ}_n, \text{Q}, \text{I} = 0; \boldsymbol{\beta}'_{\texttt{lig}}, \boldsymbol{\beta}'_{\texttt{trim}})
\end{aligned} \tag{19}
$$

where $P_{\text{annot}}(\text{delVJ}, \text{MH} \mid \text{VJ}, \text{Q}, \text{I} = 0; X, \boldsymbol{\beta}_{\texttt{lig}}, \boldsymbol{\beta}_{\texttt{trim}})$ and $P(\text{delVJ}, \text{MH} \mid \text{VJ}, \text{Q}, \text{I} = 0; \boldsymbol{\beta}'_{\texttt{lig}}, \boldsymbol{\beta}'_{\texttt{trim}})$ are defined in (18) and (17), respectively. Similarly, we define the log-likelihood function for a random sample of observed sequences $\mathcal{X}$ as:

$$
\mathcal{L}(\boldsymbol{\beta}'_{\texttt{lig}}, \boldsymbol{\beta}'_{\texttt{trim}} \mid \boldsymbol{\beta}_{\texttt{lig}}, \boldsymbol{\beta}_{\texttt{trim}}; \mathcal{X}, \text{Q}, \text{I} = 0) = \sum_{X \in \mathcal{X}} C(X) \times \ell(\boldsymbol{\beta}'_{\texttt{lig}}, \boldsymbol{\beta}'_{\texttt{trim}} \mid \boldsymbol{\beta}_{\texttt{lig}}, \boldsymbol{\beta}_{\texttt{trim}}; X, \text{Q}, \text{I} = 0) \tag{20}
$$

where $C(X)$ represents the observed count of a specific sequence $X \in \mathcal{X}$ in the sampled data and $\ell(\boldsymbol{\beta}'_{\texttt{lig}}, \boldsymbol{\beta}'_{\texttt{trim}} \mid \boldsymbol{\beta}_{\texttt{lig}}, \boldsymbol{\beta}_{\texttt{trim}}; X, \text{Q}, \text{I} = 0)$ is defined as in (19). The calculation of this expectation $\mathcal{L}$ constitutes the E-step of our EM procedure. Subsequently, in the minimization step (M-step), we update the model parameters

by minimizing the negative log-likelihood of the observed data obtained in the E-step. This minimization step is performed using gradient descent with the `JAX` and `JAXopt` packages in Python [2, 1]. The algorithm iterates between the E and M steps until changes in the negative log-likelihood between successive iterations fall below a predefined threshold, indicating convergence.

Evaluating model using simulated data

To ensure our model returned expected outputs, we designed a data simulator capable of generating data under specific microhomology regimes. The simulator first samples a V-gene and J-gene according to IGoR-derived gene usage probabilities. Next, we establish probabilities for each trimming scenario using outputs from a version of our model that excludes microhomology terms, incorporating only trimming motif and base count terms. We then adjust these trimming probabilities using a tunable parameter to simulate the effect of microhomology, and the simulator samples a trimming scenario based on these adjusted probabilities. Finally, the simulator samples a ligation scenario uniformly, unless adjusted for microhomology effects by another tunable parameter. These two tunable parameters allow us to control the influence of microhomology on trimming and ligation choices. This process generates an observed simulated sequence, and by repeating it, we obtain a large set of simulated sequences to train and evaluate our model. We ran the simulator in four modes:

1. **No microhomology effect:** Both tunable microhomology parameters set to zero; microhomology does not influence trimming or ligation choices.

2. **Microhomology affects both trimming and ligation:** Both tunable microhomology parameters set to nonzero, positive values; microhomology increases probabilities for both trimming and ligation choices.

3. **Microhomology affects trimming, but not ligation:** Trimming-related parameter set to a nonzero, positive value and ligation-related parameter set to zero; microhomology increases trimming choice probabilities but does not affect ligation probabilities.

4. **Microhomology affects ligation, but not trimming:** Ligation-related parameter set to a nonzero, positive value and trimming-related parameter set to zero; microhomology increases ligation choice probabilities but does not affect trimming probabilities.

Using these simulated datasets, we trained our model to ensure that the expected inferred parameters were obtained. We also adjusted the strength of the microhomology-related effects using these tunable parameters to ensure our model could capture these signals.

Exploring the relationship between microhomology and trimming probabilities, independent of ligation

To quantify the effect of microhomology on trimming scenario probabilities independently of ligation, we restrict our training dataset to non-productive sequences *containing* N-insertions, as their presence suggests that ligation exclusively involving germline microhomology did not occur. We aim to determine the influence of various sequence-level parameters on $P(\mathrm{delVJ} \mid \mathrm{VJ}, \mathrm{Q}, \mathrm{I} > 0)$, where delVJ represents the trimming scenario, VJ represents the gene pair, Q represents the sequence productivity, and $\mathrm{I} > 0$ represents nonzero N-insertions.

We previously demonstrated that local nucleotide identity surrounding trimming sites (the "trimming motif") and the counts of GC or AT nucleotides beyond these motifs (the "5' base-count" and "3' base-count") are highly predictive of trimming probabilities for single gene sequences [13]. Here, we model the probabilities of trimming scenarios for gene pairs using these established parameters along with a new parameter related to "intermediate microhomology." This new parameter measures the importance of the average number of microhomologous nucleotides between two trimmed sequences, which, notably, are not homologous in the final rearranged sequence, see following section for definition. A summary of these model parameters and their corresponding weights for an arbitrary gene pair $\mathrm{VJ} = (\mathrm{V}, \mathrm{J})$ and trimming scenario $\mathrm{delVJ} = (\mathrm{delV}, \mathrm{delJ})$ is given in Table S2.

Using these model features, we define a weight function f such that our model of $P(\text{delVJ}_i \mid \text{VJ}, \text{Q}, \text{I} > 0)$ will be a normalized version of this weight. We parameterize f using $\boldsymbol{\beta}$, the set of all model parameters, as follows:

$$\begin{aligned} f(\text{delVJ}, \text{VJ}; \boldsymbol{\beta}) &:= f(\text{delVJ}, \text{VJ}; \boldsymbol{\beta}_V^{\texttt{motif}}, \boldsymbol{\beta}_J^{\texttt{motif}}, \boldsymbol{\beta}_V^{\texttt{AT}}, \boldsymbol{\beta}_V^{\texttt{GC}}, \boldsymbol{\beta}_J^{\texttt{AT}}, \boldsymbol{\beta}_J^{\texttt{GC}}, \beta^{\texttt{iMH}}) \\ &:= f_{\texttt{motif}}(\text{delV}, \text{V}; \boldsymbol{\beta}_V^{\texttt{motif}}) + f_{\texttt{motif}}(\text{delJ}, \text{J}; \boldsymbol{\beta}_J^{\texttt{motif}}) \\ &\quad + f_{\texttt{count}}(\text{delV}, \text{V}; \boldsymbol{\beta}_V^{\texttt{AT}}, \boldsymbol{\beta}_V^{\texttt{GC}}) + f_{\texttt{count}}(\text{delJ}, \text{J}; \boldsymbol{\beta}_J^{\texttt{AT}}, \boldsymbol{\beta}_J^{\texttt{GC}}) \\ &\quad + f_{\texttt{iMH}}(\text{delV}, \text{delJ}, \text{V}, \text{J}; \beta^{\texttt{iMH}}). \end{aligned} \tag{21}$$

Here, the parameter-specific weights $f_{\texttt{motif}}$, $f_{\texttt{count}}$, and $f_{\texttt{iMH}}$ are summarized in Table S2 and in the following section.

With this weight formulation, we can fit a conditional logit model which posits

$$P(\text{delVJ} \mid \text{VJ}, \text{Q}, \text{I} > 0; \boldsymbol{\beta}) := \frac{\exp\big(f(\text{delVJ}, \text{VJ}; \boldsymbol{\beta})\big)}{\sum_{\text{delVJ}_n \in \mathcal{D}} \exp\big(f(\text{delVJ}_n, \text{VJ}; \boldsymbol{\beta})\big)}. \tag{22}$$

Here, VJ and delVJ are random variables representing the V-gene and J-gene pair and trimming scenario, respectively, and $\mathcal{D}$ is the set of all reasonable trimming scenarios.

The likelihood function $\ell(\boldsymbol{\beta})$ for a random sample of sequences is the likelihood of the model parameters $\boldsymbol{\beta}$ given a set of observed trimming scenarios for specific gene pairs. The log likelihood function is:

$$\begin{aligned} &\log \ell(\boldsymbol{\beta}) \\ &= \sum_{\text{VJ}_n \in \mathcal{S}} \sum_{\text{delVJ}_n \in \mathcal{D}} C(\text{delVJ}_n, \text{VJ}_n, \text{Q}, \text{I} > 0) \cdot \log P(\text{delVJ}_n \mid \text{VJ}_n, \text{Q}, \text{I} > 0; \boldsymbol{\beta}) \end{aligned} \tag{23}$$

where $\mathcal{S}$ represents the set of all gene pairs and $\mathcal{D}$ represents the set of all reasonable IGoR-inferred trimming scenarios. Here, $C(\text{delVJ}_n, \text{VJ}_n, \text{Q}, \text{I} > 0)$ is the count of sequences with IGoR-inferred trimming scenario delVJ_n, gene pair VJ_n, nonzero N-insertions, and sequence productivity Q and $P(\text{delVJ}_n \mid \text{VJ}_n, \text{Q}, \text{I} > 0; \boldsymbol{\beta})$ is given by Equation (22).

We include an additional regularization term for the intermediate-microhomology-specific parameters to help prevent over-fitting during model training. As such, we define a log loss function as

$$\mathcal{L}(\boldsymbol{\beta}) = -\log \ell(\boldsymbol{\beta}) + \lambda \cdot (\beta^{\texttt{iMH}})^2 \tag{24}$$

where $\log \ell(\boldsymbol{\beta})$ is given by (23) and λ is a L2 regularization hyperparameter. We minimize this log loss function using gradient descent with the `JAX` and `JAXopt` packages in Python [2, 1]. We use a grid search to optimize the L2 regularization hyperparameter, λ. Notably, training this model without regularization (i.e. $\lambda = 0$) yields the same results as using the `mclogit` package in R, another implementation of conditional logistic regression that does not allow for regularization.

Defining "intermediate microhomology" parameters

In addition to the previously defined parameters, we define new microhomology-related parameters to model possible intermediate effects of microhomology on the observed trimming scenario, specifically when exploring the relationship between microhomology and trimming probabilities independent of ligation (as described above). We use the term "intermediate" because these nucleotides, while not directly participating in the final ligation, may temporarily influence intermediate steps such as trimming. Let a be a non-negative integer value that represents the number of nucleotides 5' of each trimming site which are allowed to overlap between the two sequences when orienting the top strand of the V-gene sequence 5'-to-3' and the bottom strand of the J-gene sequence 3'-to-5' (e.g. as highlighted in yellow in Figure S13). Given a value of a, random variables V and J representing a V-gene and J-gene which each define a V-gene and J-gene sequence (both oriented 3'-to-5' as ordered lists), and random variables delV_i and delJ_i representing V-gene and J-gene trimming amounts (inferred directly from IGoR), the sub-sequences corresponding to this overlapping region are defined by the following ordered lists

$$\mathsf{seq}_{\mathsf{Vmh}}(\text{V}, \text{delV}_i, a) = \begin{cases} \big(\text{V}(\text{delV}_i + 2 - j)\big)_{j=(1-a)}^{0} & \text{if } a \geq 1 \\ () & \text{if } a = 0 \end{cases} \tag{25}$$

and

$$\mathtt{seq}_{\mathtt{Jmh}}(\mathrm{J}, \mathrm{delJ}_i, a) = \begin{cases} \big(\mathrm{J}(\mathrm{delJ}_i + 2 - j)\big)_{j=0}^{(1-a)} & \text{if } a \geq 1 \\ () & \text{if } a = 0. \end{cases} \tag{26}$$

Here, $\mathrm{V}(\mathrm{delV}_i + 2 - j)$ and $\mathrm{J}(\mathrm{delJ}_i + 2 - j)$ represent the nucleotide identities at a sequence position j where positions $j \leq 0$ represent sequence positions 5' of the trimming sites and positions $j > 0$ represent sequence positions 3' of the trimming sites. The resulting sub-sequences, $\mathtt{seq}_{\mathtt{Vmh}}(\mathrm{V}, \mathrm{delV}_i, a)$ and $\mathtt{seq}_{\mathtt{Jmh}}(\mathrm{J}, \mathrm{delJ} + i, a)$, are oriented in the 5'-to-3' and 3'-to-5' directions, respectively, making them complementary. To quantify microhomology, we can define a function g which will count the number of complementary (i.e. microhomologous) nucleotides between two arbitrary overlapping, equal-length sequence regions, x and y, as

$$g(x, y) = \sum_{i=0}^{\mathrm{len}(x)} \begin{cases} 1 & \text{if } x(i) \text{ is complementary to } y(i) \\ 0 & \text{otherwise.} \end{cases} \tag{27}$$

It has been established that classical non-homologous end joining, which is the joining process used during V(D)J recombination, may involve up to four nucleotides of microhomology [7]. As such, we quantify the average number of non-contiguous microhomologous nucleotides across these overlapping interior sub-sequences corresponding to each $a \in \{1, 2, 3, 4\}$ as follows:

$$m(\mathrm{V}, \mathrm{J}, \mathrm{delV}_i, \mathrm{delJ}_i) := \frac{\sum_{a \in \{1,2,3,4\}} g\big(\mathtt{seq}_{\mathtt{Vmh}}(\mathrm{V}, \mathrm{delV}_i, a), \mathtt{seq}_{\mathtt{Jmh}}(\mathrm{J}, \mathrm{delJ}_i, a)\big)}{4} \tag{28}$$

With this average number of microhomologous nucleotides, we define an *intermediate microhomology* model parameter, $\beta^{\mathtt{iMH}}$, and a corresponding weight function for a pair of IGoR-inferred trimming sites delV_i and delJ_i and genes V and J:

$$f_{\mathtt{iMH}}(\mathrm{delV}_i, \mathrm{delJ}_i, \mathrm{V}, \mathrm{J}; \beta^{\mathtt{iMH}}) := \beta^{\mathtt{iMH}} \cdot m(\mathrm{V}, \mathrm{J}, \mathrm{delV}_i, \mathrm{delJ}_i) \tag{29}$$

using the previously defined sequences and the function m as defined in (28).

2 Supporting Figures and Tables

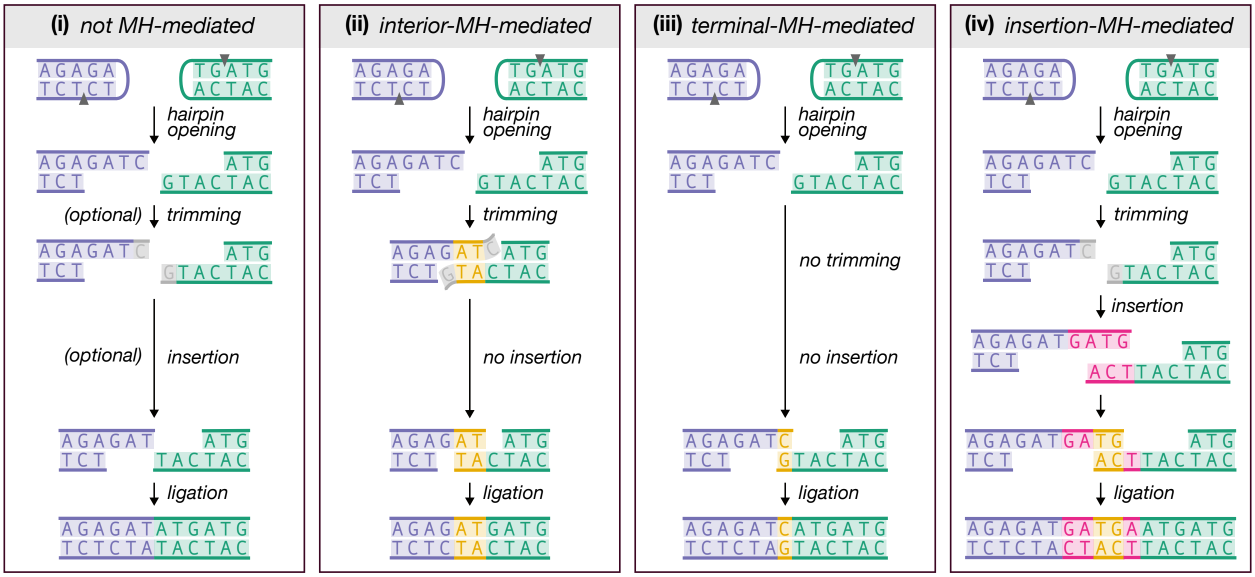

Figure S1: Illustration of how microhomologous nucleotides could influence trimming and/or ligation during V(D)J recombination. The example depicts microhomologous regions (yellow), trimmed nucleotides (gray), and inserted nucleotides (pink) for a V-gene (purple) and J-gene (green), highlighting four possible regimes: (i) no microhomology influence, (ii) influence of interior microhomology, (iii) influence of terminal microhomology, and (iv) influence of insertion-dependent microhomology. Terminal and interior microhomologous nucleotides are germline-encoded, whereas insertion-dependent microhomologous nucleotides are randomly added by terminal deoxynucleotidyl transferase (TdT) and are not encoded in the germline. Germline-encoded microhomologous regions are classified as terminal microhomology when they are located at the ends of untrimmed sequences, and as interior microhomology when they are located within the interior regions of the gene sequences.

Table S1: Summary of all notation used in our modeling.

Variable	Description
**General notation**	
X	arbitrary sampled sequence
$\mathcal{X}$	set of sampled sequences
V, J	random variables for the V- and J-gene
VJ	ordered pair of genes, (V, J)
I	random variable for number of N-insertions
Q	deterministic variable for sequence productivity
MH	random variable for number of microhomologous nucleotides within the observed sequence; also referred to as a “ligation scenario”
$\mathrm{delV}, \mathrm{delJ}$	random variables for trimming amounts from V/J-gene

delVJ	pair of trimming amounts, (delV, delJ); also referred to as a "trimming scenario"
$\mathrm{delV}_i, \mathrm{delJ}_i$	random variables for nucleotides deleted from the V/J-gene, inferred directly by IGoR
A_X	set of possible trimming and ligation annotations for a sequence X
**Motif parameter notation**	
$\boldsymbol{\beta}_V^{\texttt{motif}}, \boldsymbol{\beta}_J^{\texttt{motif}}$	set of all V/J-gene motif parameters, defined in detail within Supplementary Materials
$f_{\texttt{motif}}(\mathrm{delV}, \mathrm{V}; \boldsymbol{\beta}_V^{\texttt{motif}})$	V-gene motif weight function (5)
$f_{\texttt{motif}}(\mathrm{delJ}, \mathrm{J}; \boldsymbol{\beta}_J^{\texttt{motif}})$	J-gene motif weight function (5)
**Base count parameter notation**	
$\boldsymbol{\beta}_V^{\texttt{AT}}$, $\boldsymbol{\beta}_V^{\texttt{GC}}$	V-gene AT/GC base count parameters, defined in detail within Supplementary Materials
$\boldsymbol{\beta}_J^{\texttt{AT}}$, $\boldsymbol{\beta}_J^{\texttt{GC}}$	J-gene AT/GC base count parameters
$f_{\texttt{count}}(\mathrm{delV}, \mathrm{V}; \boldsymbol{\beta}_V^{\texttt{AT}}, \boldsymbol{\beta}_V^{\texttt{GC}})$	V-gene base count weight function (10)
$f_{\texttt{count}}(\mathrm{delJ}, \mathrm{J}; \boldsymbol{\beta}_J^{\texttt{AT}}, \boldsymbol{\beta}_J^{\texttt{GC}})$	J-gene base count weight function (10)
**Microhomology parameter notation**	
$\beta^{\texttt{trimMH}}, \beta^{\texttt{ligMH}}$	trimming/ligation microhomology parameters
$f_{\texttt{trimMH}}(\mathrm{delVJ}, \mathrm{VJ}; \beta^{\texttt{trimMH}})$	trimming-related microhomology weight function (11)
$f_{\texttt{ligMH}}(\mathrm{MH}; \beta^{\texttt{ligMH}})$	ligation-related microhomology weight function (12)
**Model notation**	
$\boldsymbol{\beta}_{\texttt{trim}}$	set of all trimming-related model parameters: $\boldsymbol{\beta}_V^{\texttt{motif}}, \boldsymbol{\beta}_J^{\texttt{motif}}, \boldsymbol{\beta}_V^{\texttt{AT}}, \boldsymbol{\beta}_J^{\texttt{AT}}, \boldsymbol{\beta}_V^{\texttt{GC}}, \boldsymbol{\beta}_J^{\texttt{GC}}, \beta^{\texttt{trimMH}}$
$\boldsymbol{\beta}_{\texttt{lig}}$	set of all ligation-related model parameters: $\beta^{\texttt{ligMH}}$
$f_{\texttt{trim}}(\mathrm{delVJ}, \mathrm{VJ}; \boldsymbol{\beta}_{\texttt{trim}})$	trimming-related weight function (2)
$f_{\texttt{lig}}(\mathrm{delVJ}, \mathrm{MH}, \mathrm{VJ}; \boldsymbol{\beta}_{\texttt{lig}})$	ligation-related weight function (3)
$P(\mathrm{delVJ}, \mathrm{MH} \mid \mathrm{VJ}, \mathrm{Q}, \mathrm{I} = 0; \boldsymbol{\beta}_{\texttt{trim}}, \boldsymbol{\beta}_{\texttt{lig}})$	two-step conditional logit model (17)
$P_{\mathrm{annot}}(\mathrm{delVJ}, \mathrm{MH} \mid \mathrm{VJ}, \mathrm{Q}, \mathrm{I} = 0; X, \boldsymbol{\beta}_{\texttt{lig}}, \boldsymbol{\beta}_{\texttt{trim}})$	model-derived trimming and ligation annotation probability (18)
$\ell(\boldsymbol{\beta}'_{\texttt{lig}}, \boldsymbol{\beta}'_{\texttt{trim}} \mid \boldsymbol{\beta}_{\texttt{lig}}, \boldsymbol{\beta}_{\texttt{trim}}; X, \mathrm{Q}, \mathrm{I} = 0)$	expected log-likelihood for single sequence (19)
$\mathcal{L}(\boldsymbol{\beta}'_{\texttt{lig}}, \boldsymbol{\beta}'_{\texttt{trim}} \mid \boldsymbol{\beta}_{\texttt{lig}}, \boldsymbol{\beta}_{\texttt{trim}}; \mathcal{X}, \mathrm{Q}, \mathrm{I} = 0)$	log-likelihood for observed sequences (20)

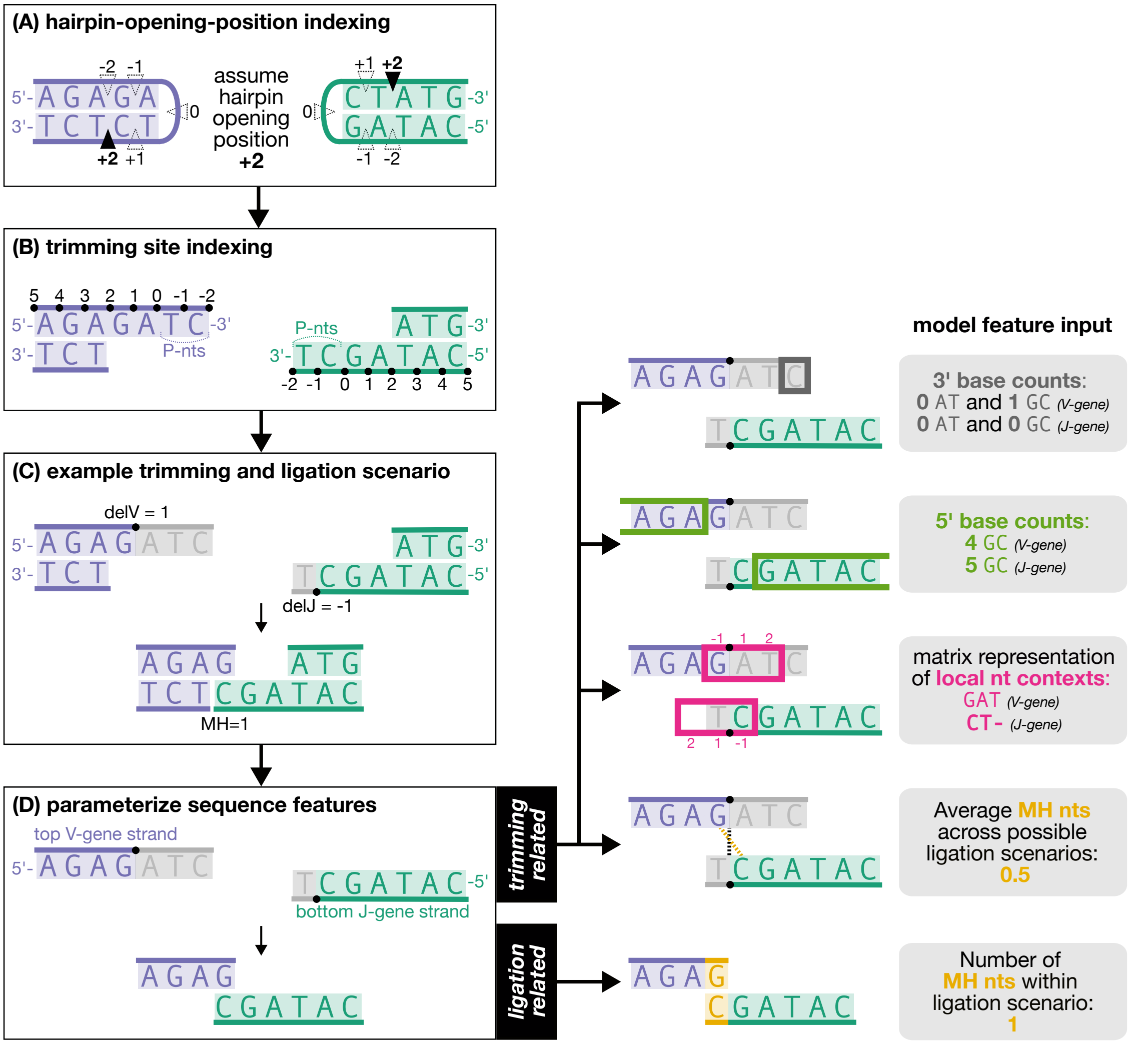

Figure S2: Overview of how sequence are transformed into features for regression. **(A)** During early stages of V(D)J recombination, the hairpin of each gene is opened. Hairpins are most frequently opened at position +2, but other positions are possible. For modeling, we assume all hairpins open at +2. **(B)** Hairpin opening creates a 4-nucleotide single-stranded overhang, with 2 nucleotides considered P-nucleotides. Each gene can then undergo nucleotide trimming. Trimming sites are indexed such that negative values represent P-nucleotide deletions, while positive values represent coding sequence deletions. For example, a deletion of 0 trims to the end of the germline gene (removing 2 P-nucleotides), and -2 indicates no trimming of P-nucleotides or gene sequence. This indexing is consistent with the IGoR software [10]. **(C)** In this example trimming and ligation scenario, the V-gene is trimmed by 3 nucleotides (V-trimming site delV $= 1$), and the J-gene is trimmed by 1 nucleotide (J-trimming site, delJ $= -1$). The trimmed genes are ligated with 1 nucleotide of microhomology. **(D)** Illustration of sequence features and their alignment for an arbitrary V- and J-gene pair and example trimming and ligation scenario. For modeling, only the top strand of the V-gene and the bottom strand of the J-gene are considered, consistent with the most common overhang polarities. Features related to ligation include the microhomology-related feature (yellow), which captures the number of microhomologous nucleotides in the ligation scenario. Features related to trimming include the microhomology-related feature (yellow), which captures the average number of microhomologous nucleotides across all possible ligation scenarios for the given trimming scenario; the trimming motif features (pink), which represent the identities of nucleotides adjacent to the trimming site and are indexed relative to the site, with negative indices indicating positions 5’ of the site and positive indices indicating positions 3’; the 5’ base count parameters (green), which capture the GC nucleotide count within 10 nucleotides upstream of the trimming motif; and the 3’ base count parameters (gray), which capture the GC and AT nucleotide counts downstream of the trimming motif, if applicable.

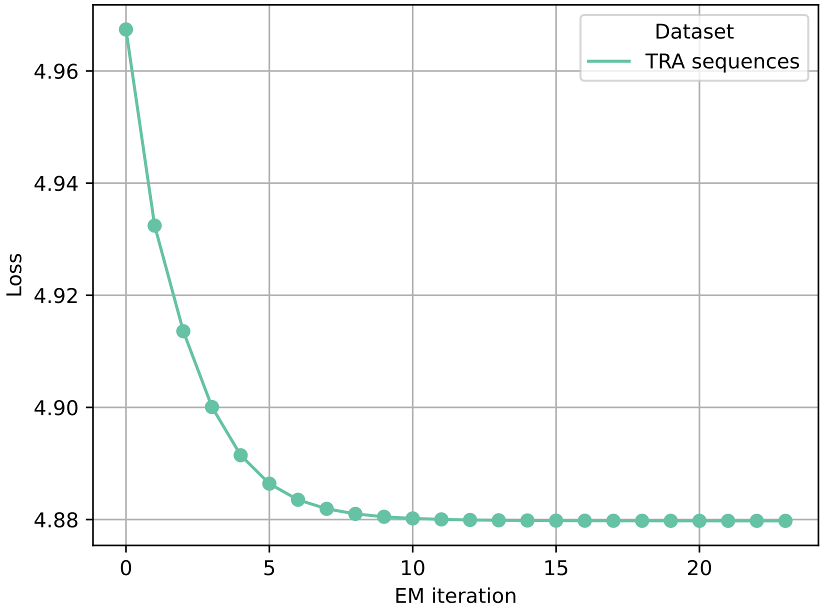

Figure S3: Convergence of the expectation-maximization (EM) algorithm using a training dataset of non-productive TCRα sequences without N-insertions, alongside their corresponding sets of potential trimming and ligation scenario annotations (as detailed in Methods). The y-axis represents the expected per-sequence log loss, as defined in (20).

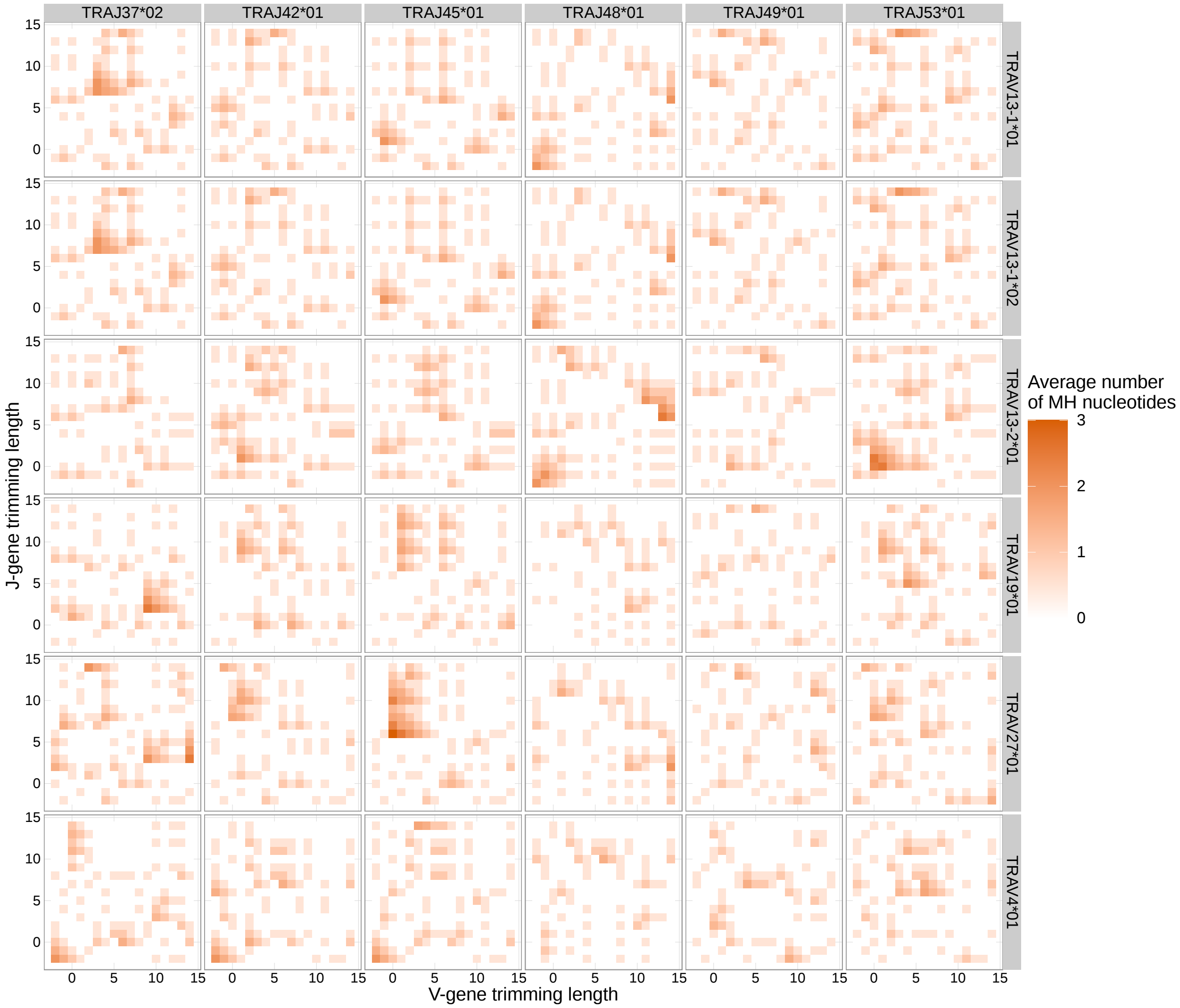

Figure S4: The distribution of complementary sequence regions capable of forming microhomologous regions during V(D)J recombination varies by trimming amounts and V-J gene pairs. Depending on the gene pair, there is potential for both interior and terminal microhomology (MH). For instance, the TRAV13-1*01 and TRAJ48*01 gene pair shows potential for terminal microhomology (e.g. the average number of MH nucleotides for the untrimmed sequences—both genes trimmed at the -2 site—is nonzero), as well as interior microhomology. In contrast, the TRAV13-1*01 and TRAJ37*02 gene pair lacks terminal microhomology potential (e.g. the average number of MH nucleotides for the untrimmed sequences is zero) but has an abundance of interior microhomology. The average microhomology counts are calculated across all possible ligation scenarios for each gene pair trimming scenario. Only the most frequently used gene pairs are plotted here.

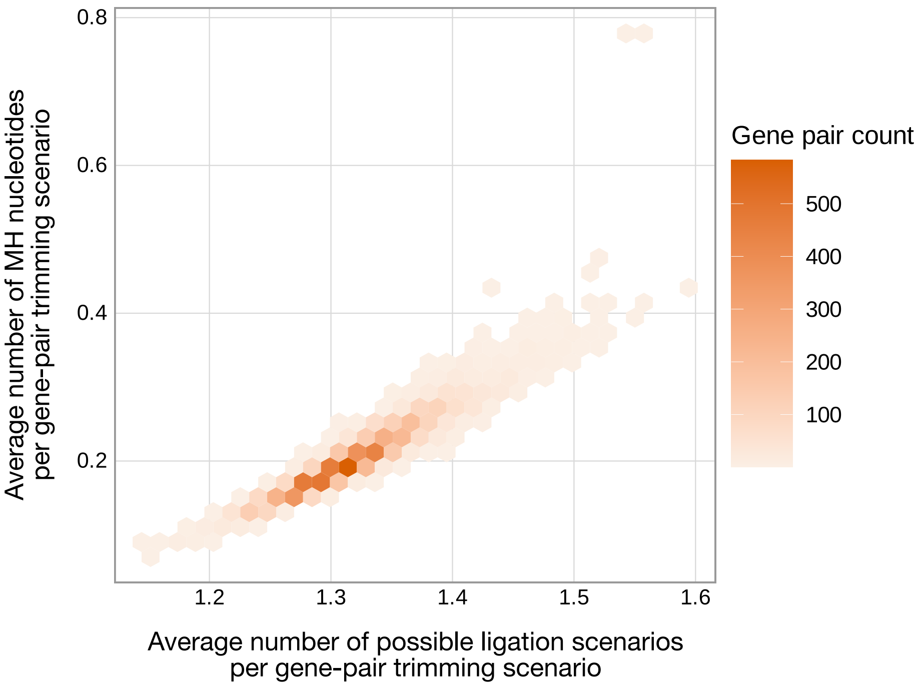

Figure S5: Complementary sequence regions capable of forming microhomologous regions during V(D)J recombination are common between germline V- and J-genes in the *TRA* locus. As the average number of microhomologous nucleotides increases for a given gene pair trimming scenario, so does the average number of possible ligation scenarios. The median average number of microhomologous nucleotides across all possible gene pair trimming scenarios is 0.1978, corresponding to a median of 1.3149 possible ligation scenarios. Since a median of exactly one ligation scenario would indicate that all scenarios involve zero microhomology, this suggests that most trimming scenarios result in multiple ligation outcomes—both with and without microhomology. The average values are calculated across all trimming scenarios for each gene pair, with each gene pair plotted only once.

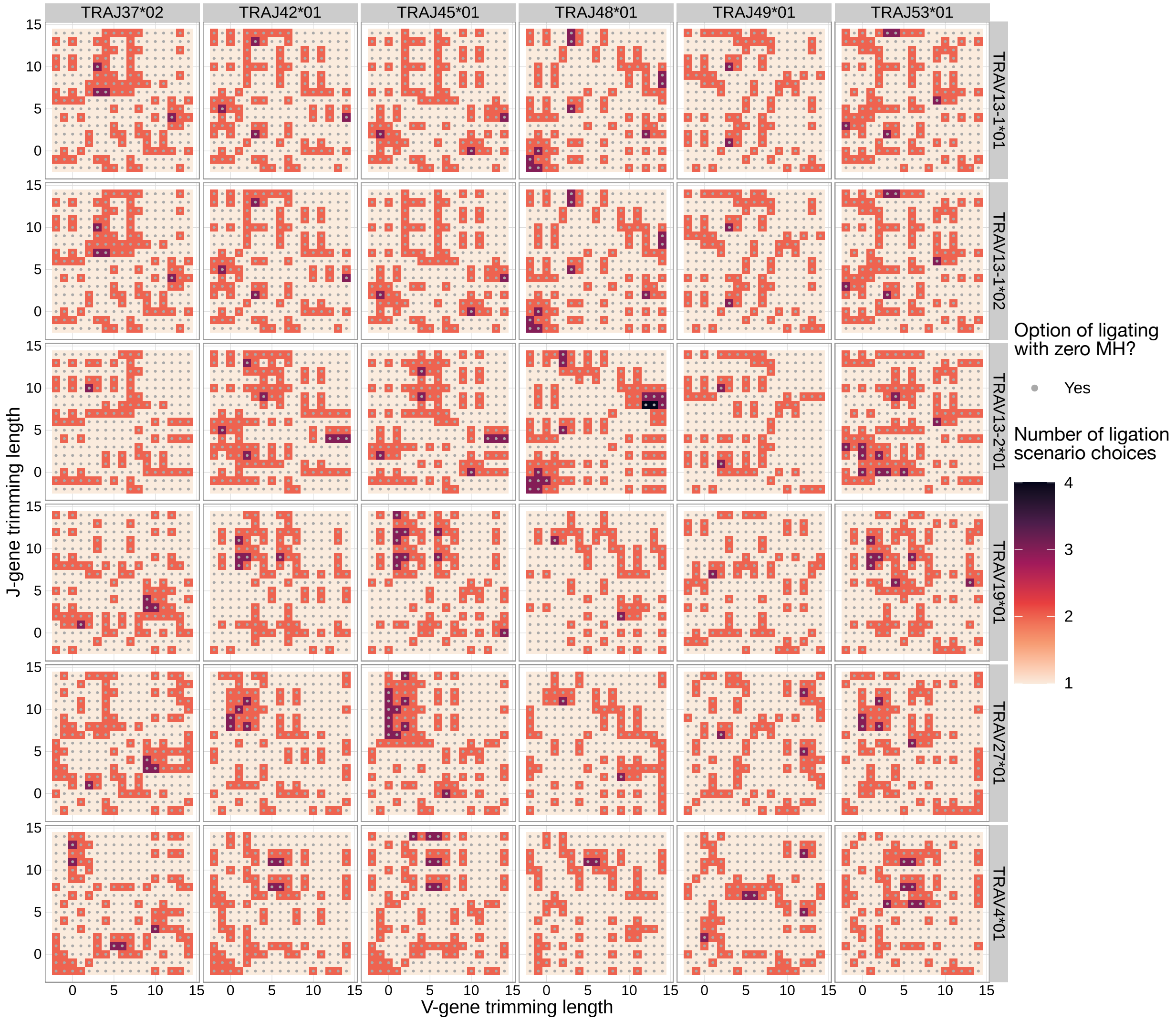

Figure S6: The distribution of trimming scenarios with multiple ligation options (e.g. varying amounts of microhomology in the observed sequence) varies across V-J gene pairs. All gene pair trimming scenarios can ligate with zero nucleotides of microhomology (indicated by gray dots in the plot), providing at least one ligation scenario choice. Depending on the gene pair, there may be potential for both interior- and terminal-microhomology-mediated trimming and ligation (e.g. ligation using nonzero microhomology), which would increase the number of possible ligation scenario choices. For example, the TRAV13-1*01 and TRAJ48*01 gene pair shows potential for terminal-microhomology-mediated ligation (e.g. ligating the untrimmed sequences—both genes trimmed at the -2 site—using nonzero microhomology), as well as interior-microhomology-mediated ligation. In contrast, the TRAV13-1*01 and TRAJ37*02 gene pair lacks potential for terminal-microhomology-mediated ligation (e.g. the untrimmed sequences can only be ligated using zero microhomology) but has substantial potential for interior-microhomology-mediated ligation. Only the most frequently used gene pairs are plotted here.

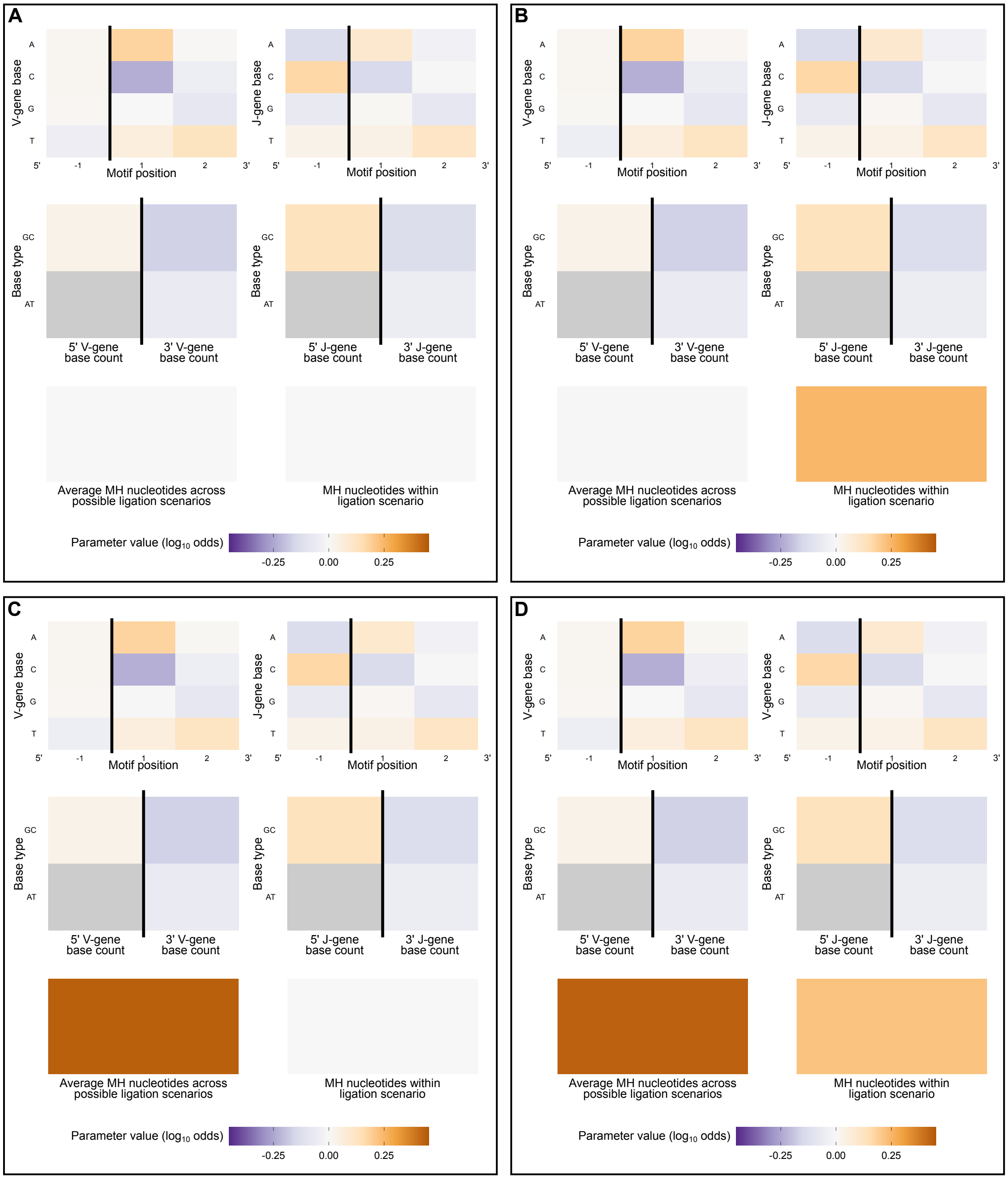

Figure S7: Parameters inferred from simulated data emulating varying levels of microhomology involvement in V(D)J recombination trimming and ligation (see Supplementary Materials). The model was trained using four different simulated datasets. (**A**) Parameters inferred from data where microhomology does not influence trimming or ligation choices. In this scenario, the microhomology-related parameters show no signal. (**B**) Parameters inferred from data where microhomology increases ligation probabilities but does not affect trimming probabilities. Here, the trimming-related microhomology parameter shows no signal, while the ligation-related parameter shows a strong positive signal. (**C**) Parameters inferred from data where microhomology increases trimming probabilities but does not affect ligation probabilities. In this case, the ligation-related microhomology parameter shows no signal, while the trimming-related parameter shows a strong positive signal. (**D**) Parameters inferred from data where microhomology increases both trimming and ligation probabilities. As expected, both microhomology-related parameters exhibit strong positive signals. The patterns observed in this simulated dataset closely matches those inferred from actual data. All trimming motif and base count parameters remain consistent across all simulated datasets.

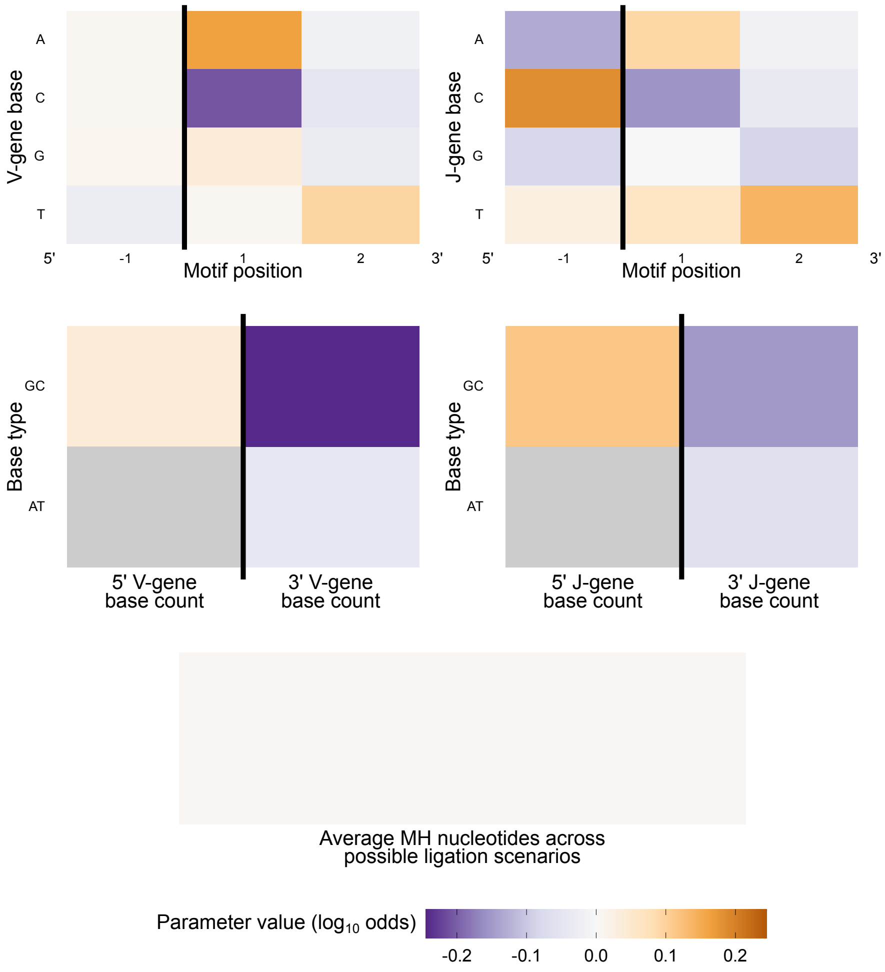

Figure S8: Parameters inferred from a model trained on sequences with N-insertions, which lack germline-dependent ligation. Due to the unknown composition of inserted nucleotides prior to ligation, ligation patterns cannot be detected, allowing this model to specifically explore trimming probabilities independent of ligation (see Supplementary Materials). The ligation-related microhomology parameter was excluded from this model. Inferred trimming motif and base count parameters align with previous analyses of individual V- and J-gene sequences [13]. The inferred trimming-related microhomology parameter indicates that microhomology has only a minimal effect on trimming probabilities, suggesting a limited independent role of microhomology in trimming.

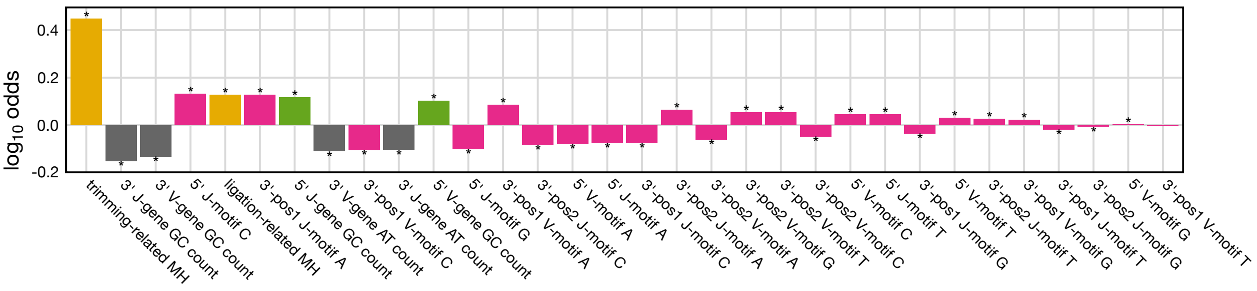

Figure S9: Both MH-related parameters (gold) exhibit large effect sizes, with the trimming-related MH parameter showing the strongest positive effect. Stars indicate parameters significant at a Bonferroni-corrected threshold of 0.0016.

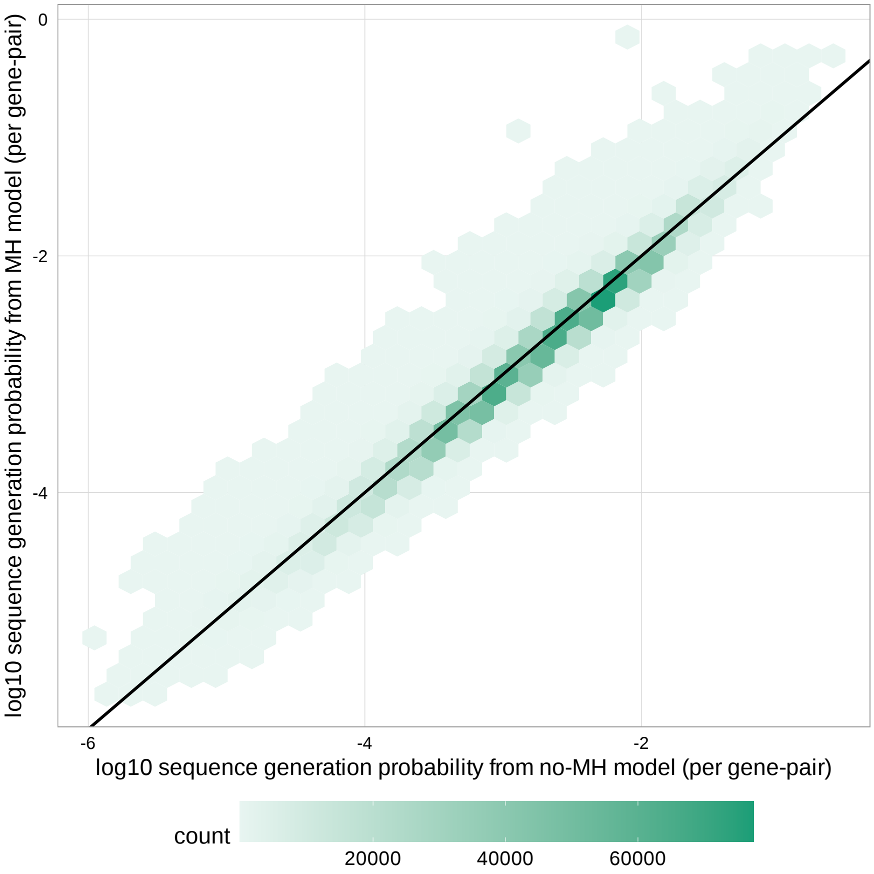

Figure S10: Sequence generation probabilities differ slightly between models that include microhomology effects and those that do not. Both the model parameterizing microhomology (MH model) and the one that does not (no-MH model) include trimming motif and base-count parameters and are identical except for the inclusion of microhomology terms. For each model, we calculate sequence generation probabilities as the aggregated probability of all possible trimming and ligation scenarios for a sequence, normalized across all sequences for a given V-J pair.

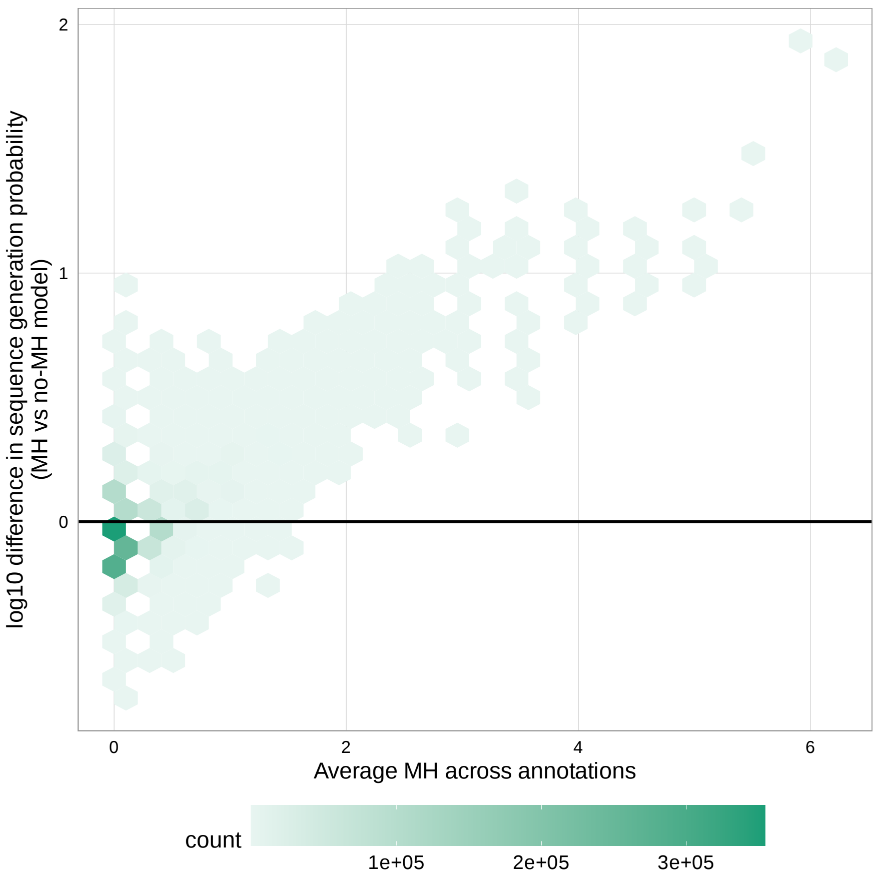

Figure S11: As the average number of microhomologous nucleotides across possible annotation scenarios for a given sequence increases, the difference in sequence generation probabilities between the model parameterizing microhomology (MH model) and the one that does not (no-MH model) becomes larger. Both models include trimming motif and base-count parameters and are identical except for the inclusion of microhomology terms. The plotted differences are calculated using probabilities normalized across all possible sequences for each V-J gene pair, ensuring the sum of these differences is zero.

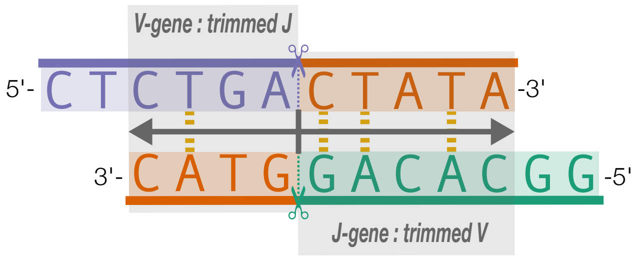

Figure S12: Cartoon showing the alignment of V-gene (purple) and J-gene (green) sequences at their inferred trimming sites (marked with scissors) without gaps. Trimmed regions of each sequence are shown in orange. We focus on two regions: (1) overlap between V-gene and trimmed J-gene (V-$gene$:$trimmed$-J) and (2) overlap between J-gene and trimmed V-gene (J-$gene$:$trimmed$-V). The function h in (1) counts contiguous, complementary nucleotides from the aligned trimming site (indexed as zero). Arrows show the counting direction for each region. Contiguous, complementary nucleotides are counted only if adjacent to the trimming site. In this example, V-$gene$:$trimmed$-J has 0 contiguous complementary nucleotides, and J-$gene$:$trimmed$-V has 2.

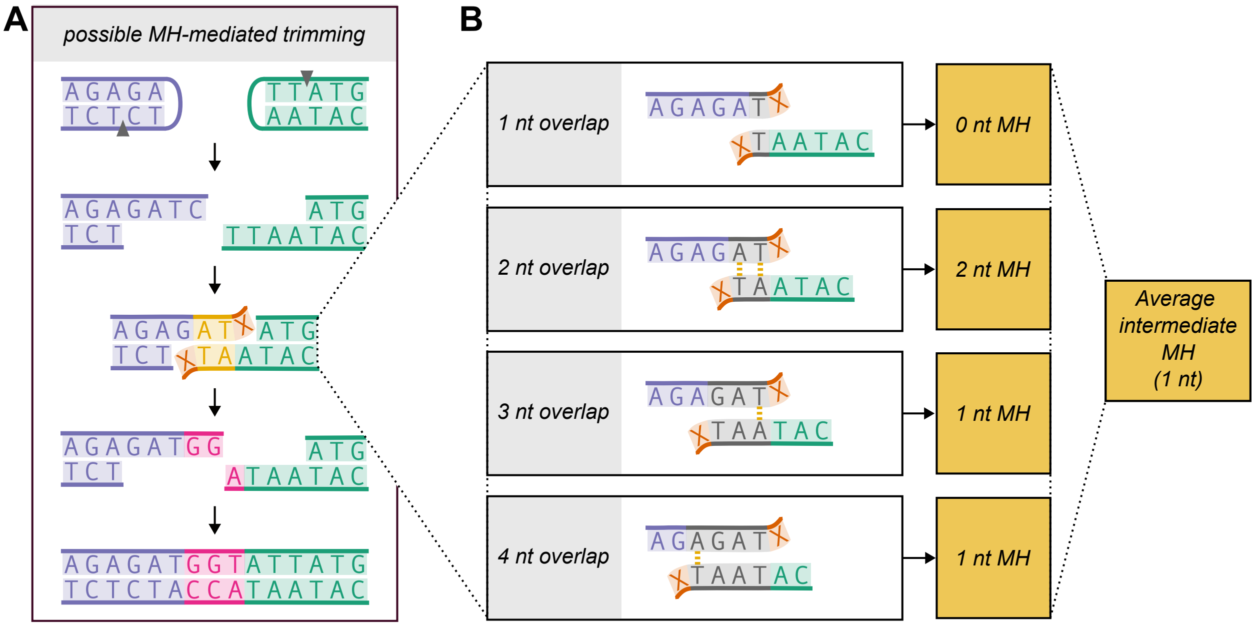

Figure S13: (**A**) Diagram illustrating V(D)J recombination steps, emphasizing possible internal/intermediate microhomology during trimming. This intermediate microhomology may occur in both single-stranded overhangs and double-stranded regions due to sequence breathing. The final joined sequence, post-N-insertion (pink), is shown in the last box. (**B**) Definition of overlapping sequence regions for specified V-gene, J-gene, and trimming scenario. The top strand of a V-gene and the bottom strand of a J-gene are each shown with one nucleotide removed (trimmed nucleotides in orange). Overlapping regions (highlighted in gray) are obtained by aligning the sequences such that an integer value, a, of nucleotides 5' of each trimming site overlap. The value of a ranges from 1 to 4 nucleotides. Despite what is shown in this example, two genes can be trimmed by different amounts and still yield these overlapping regions. Complementary nucleotides in these regions are indicated by vertical yellow lines. The final joined sequence post-trimming and insertion is shown in the last box of panel (A).

Table S2: Summary of all parameters and parameter-specific weights for an arbitrary gene pair $\mathrm{VJ} = (\mathrm{V}, \mathrm{J})$ and trimming scenario $\mathrm{delVJ} = (\mathrm{delV}, \mathrm{delJ})$. Detailed definitions of each parameter and corresponding weights are located within the Supplementary Materials.

**Parameter**	**Description**	**Notation**	**Parameter weight**
V-gene motif parameters	parameterizes the importance of several nucleotides on either side of the V-gene trimming site	$\boldsymbol{\beta}_V^{\texttt{motif}}$	$f_{\texttt{motif}}(\mathrm{delV}, \mathrm{V}; \boldsymbol{\beta}_V^{\texttt{motif}})$ (5)
J-gene motif parameters	parameterizes the importance of several nucleotides on either side of the J-gene trimming site	$\boldsymbol{\beta}_J^{\texttt{motif}}$	$f_{\texttt{motif}}(\mathrm{delJ}, \mathrm{J}; \boldsymbol{\beta}_J^{\texttt{motif}})$ (5)
V-gene base count parameters	parameterizes the importance of the counts of GC and AT nucleotides beyond the V-gene trimming motif	$\boldsymbol{\beta}_V^{\texttt{AT}}$ and $\boldsymbol{\beta}_V^{\texttt{GC}}$	$f_{\texttt{count}}(\mathrm{delV}, \mathrm{V}; \boldsymbol{\beta}_V^{\texttt{AT}}, \boldsymbol{\beta}_V^{\texttt{GC}})$ (10)
J-gene base count parameters	parameterizes the importance of the counts of GC and AT nucleotides beyond the J-gene trimming motif	$\boldsymbol{\beta}_J^{\texttt{AT}}$ and $\boldsymbol{\beta}_J^{\texttt{GC}}$	$f_{\texttt{count}}(\mathrm{delJ}, \mathrm{J}; \boldsymbol{\beta}_J^{\texttt{AT}}, \boldsymbol{\beta}_J^{\texttt{GC}})$ (10)
inter-mediate microhomol-ogy parameters	parameterizes the importance of the average number of non-contiguous intermediate microhomology between a gene pair given a trimming scenario	$\beta^{\texttt{iMH}}$	$f_{\texttt{iMH}}(\mathrm{delV}, \mathrm{delJ}, \mathrm{V}, \mathrm{J}; \beta^{\texttt{iMH}})$ (29)
trimming-related observed mi-crohomology parameters	parameterizes the importance of the average number of contiguous microhomology across all possible ligation scenarios for a given trimming scenario and gene pair	$\beta^{\texttt{trimMH}}$	$f_{\texttt{trimMH}}(\mathrm{delVJ}, \mathrm{VJ}; \beta^{\texttt{trimMH}})$ (11)
ligation-related observed mi-crohomology parameters	parameterizes the importance of the number of contiguous microhomologous nucleotides for a given ligation scenario, trimming scenario, and gene pair	$\beta^{\texttt{ligMH}}$	$f_{\texttt{ligMH}}(\mathrm{MH}; \beta^{\texttt{ligMH}})$ (12)

3 SI References

[1] Mathieu Blondel, Quentin Berthet, Marco Cuturi, Roy Frostig, Stephan Hoyer, Felipe Llinares-López, Fabian Pedregosa, and Jean-Philippe Vert. Efficient and modular implicit differentiation. *arXiv [cs.LG]*, May 2021.

[2] James Bradbury, Roy Frostig, Peter Hawkins, Matthew James Johnson, Chris Leary, Dougal Maclaurin, George Necula, Adam Paszke, Jake VanderPlas, Skye Wanderman-Milne, and Qiao Zhang. JAX: composable transformations of Python+NumPy programs, 2018.

[3] G H Gauss and M R Lieber. Mechanistic constraints on diversity in human V(D)J recombination. *Mol. Cell. Biol.*, 16(1):258–269, January 1996.

[4] Nelli Heikkilä, Silja Sormunen, Joonatan Mattila, Taina Härkönen, Mikael Knip, Emmi-Leena Ihantola, Tuure Kinnunen, Ilkka P Mattila, Jari Saramäki, and T Petteri Arstila. Generation of self-reactive, shared T-cell receptor alpha chains in the human thymus. *J. Autoimmun.*, 119(102616):102616, May 2021.

[5] Nelli Heikkilä, Reetta Vanhanen, Dawit A Yohannes, Iivari Kleino, Ilkka P Mattila, Jari Saramäki, and T Petteri Arstila. Human thymic T cell repertoire is imprinted with strong convergence to shared sequences. *Mol. Immunol.*, 127:112–123, November 2020.

[6] Katherine J L Jackson, Bruno Gaeta, William Sewell, and Andrew M Collins. Exonuclease activity and P nucleotide addition in the generation of the expressed immunoglobulin repertoire. *BMC Immunol.*, 5:19, September 2004.

[7] Michael R Lieber. The mechanism of double-strand DNA break repair by the nonhomologous DNA end-joining pathway. *Annu. Rev. Biochem.*, 79:181–211, 2010.

[8] Haihui Lu, Klaus Schwarz, and Michael R Lieber. Extent to which hairpin opening by the Artemis:DNA-PKcs complex can contribute to junctional diversity in V(D)J recombination. *Nucleic Acids Res.*, 35(20):6917–6923, October 2007.

[9] Yunmei Ma, Ulrich Pannicke, Klaus Schwarz, and Michael R Lieber. Hairpin opening and overhang processing by an Artemis/DNA-dependent protein kinase complex in nonhomologous end joining and V(D)J recombination. *Cell*, 108(6):781–794, March 2002.

[10] Quentin Marcou, Thierry Mora, and Aleksandra M Walczak. High-throughput immune repertoire analysis with IGoR. *Nat. Commun.*, 9(1):561, February 2018.

[11] B Nadel and A J Feeney. Nucleotide deletion and P addition in V(D)J recombination: a determinant role of the coding-end sequence. *Mol. Cell. Biol.*, 17(7):3768–3778, July 1997.

[12] H Robins and O Pearson. Normal human PBMC, deep sequencing, TCRB vs TCRG comparison, April 2015. Accessed: 2022-10-25.

[13] Magdalena L Russell, Noah Simon, Philip Bradley, and Frederick A Matsen, 4th. Statistical inference reveals the role of length, GC content, and local sequence in V(D)J nucleotide trimming. *Elife*, 12, May 2023.

[14] Santosh K Srivastava and Harlan S Robins. Palindromic nucleotide analysis in human T cell receptor rearrangements. *PLoS One*, 7(12):e52250, December 2012.
